# In Vitro Screening of the Antifungal and Antimycotoxin Effects of a Stilbenoids-Riche Grapevine Cane Extract on *Fusarium graminearum*, *Aspergillus flavus* and *Penicillium expansum*

**DOI:** 10.3390/toxins17090454

**Published:** 2025-09-09

**Authors:** Dorian Aznar, Alexandre Colas de la Noue, Luc P. R. Bidel, Caroline Cayzac, Charlie Poss, Eloïse Ciordia, Andréa Cozette, Angélique Fontana, Fanny Rolet, Caroline Strub

**Affiliations:** 1 Qualisud, Univ Montpellier, CIRAD, Institut Agro, IRD, Avignon Univ, Univ de La Réunion, 4 CIRAD, UMR QualiSud, F-34398 Montpellier, France; charlie.poss@cirad.fr (C.P.); angelique.fontana@umontpellier.fr (A.F.); caroline.strub@umontpellier.fr (C.S.); 2 Antofénol, Place Eugène Bataillon, Bldg. 15, 3rd Floor, F-34095 Montpellier, France; c.cayzac@antofenol.com (C.C.); e.ciordia@antofenol.com (E.C.); a.cozette@antofenol.com (A.C.); f.rolet@antofenol.com (F.R.); 3 UMR IATE, University of Montpellier, CIRAD, INRAE, Institut Agro, F-34398 Montpellier, France; luc.bidel@umontpellier.fr; 4 CIRAD, UMR Qualisud, F-34398 Montpellier, France

**Keywords:** polyphenols, *F. graminearum*, *A. flavus*, *P. expansum*, trichotecenes, aflatoxins, patulin, grapevine cane, biocontrol

## Abstract

Grapevine cane, an abundant viticultural by-product, contains high levels of stilbenoids and therefore holds promise as a natural antifugal and antimycotoxigenic agent. Produced by a microwave-assisted hydro-ethanolic extraction process, the grapevine cane extract (GCE) was tested for its activity against three mycotoxigenic fungi *F. graminearum*, *A. flavus*, and *P. expansum*. Dose-response assays were performed, based on radial growth and inhibition of specific mycotoxin production. For all fungi, growth inhibition IC_50_ values clustered between 1.0 and 5.0 g/L, while for specific toxin production, IC_50_ were lower (≈0.5 g/L) except for patulin, which increased in a dose-dependent manner in the presence of the extract. Specific experiments were designed to highlight the effect of the extracts at various stages of the fungal life cycle (e.g., spore germination, early mycelium, and established colonies). *F. graminearum* spores’ germination was strongly inhibited (5.0 to 15 g/L), while for other fungi, germination was only delayed. Interestingly, antifungal and especially antimycotoxigenic effects were shown to be persistent after exposure.

## 1. Introduction

Filamentous fungi, including those producing mycotoxins, rank among the most significant threats to global crop production from both health and economic perspectives [1,2]. Some of the most reported species responsible for infecting crops and producing mycotoxins are *Fusarium graminearum* with type B trichothecenes (TCTBs), *Aspergillus flavus* with aflatoxins (AFLAs), and *Penicillium expansum* with patulin (PAT) [3,4,5]. TCTBs include deoxynivalenol (DON) and its acetylated forms 3-acetyl-4-deoxynivalenol and 15-acetyl-4-deoxynivalenol (3-ADON and 15-ADON) and AFLAs include aflatoxin B1 (AFB_1_)–B2 (AFB_2_)–G1 (AFG_1_)–G_2_ (AFG2). These mycotoxins encompass a broad range of toxic secondary metabolites known for their potential genotoxic and carcinogenic effects on human health [6,7]. Numerous factors contribute to fungal development and mycotoxin accumulation in crops—both before harvest and during storage or processing—such as climatic conditions, temperature, water activity (a_w_), nutrient availability, insect infestation, specific microorganism interactions, harvest timing, drying methods or inadequate storage practices [8,9,10]. Mycotoxin regulations for agricultural products have been estalished in many countries worldwide, with strict limits set specifically for each agricultural commodity [11,12,13]. Despite intensified fungal surveillance and mycotoxins control, the Food and Agriculture Organization (FAO) estimated that at least 25% of global crops contain mycotoxins above detectable levels, resulting in an annual loss of approximately one billion metric tons of agricultural products. In addition, according to the Rapid Alert System for Food and Feed (RASFF), mycotoxins are consistently identified as one of the top ten hazards in food products, particularly in cereals and nuts. The damage caused by fungi in the field and after harvest, combined with mycotoxin contaminations, significantly impacts agricultural production and the economy due to the rejection of contaminated commodities [14,15]. Several strategies have been developed to control fungal and mycotoxins contamination at both, pre-harvest and post-harvest stages [16]. Pre-harvest approaches focus on preventing fungal development and mycotoxins production in the field, while post-harvest approaches focus on monitoring toxins presence at key control points and mycotoxin decontamination [17,18,19]. Thus, the use of fungicides remains central to prevent the proliferation of mycotoxin-producing fungi in crops. However, even used in accordance with optimal agronomic practices, these plant protection products may negatively affect human health and the environment, and residues may persist on crops. In 2009, the authorization of all the active substances used in European Union for plant protection was reevaluated, leading to national action plans in order to reduce the impact of pesticides. Along with their detrimental effects, extensive use of pesticides have favoured the emergence of drug-resistant fungal strains [20].

In this context, the search for new active substances and eco-friendly fungicides has become a priority and the development of biopesticides appears to be an encouraging approach. Biopesticides solutions include macro-, microorganism, natural substances, and a chemical mediator [21]. Altogether, combined to each other, and associated to good agricultural practices, biopesticides represent a suitable alternative for crop protection. Plant-based extracts are promising due to their diverse antifungal compounds, lower toxicity to human health and the environment and their biodegradability. In addition, compared to single, purified molecules, natural plant extracts may offer synergistic benefits, as multiple active compounds can target various fungal physiological pathways, thereby boosting efficacy and limiting the emergence of resistant strains [22,23,24]. Beyond their direct phytochemical effects, plant-derived residues might be used as a substrate to grow biocontrol microorganisms that produces antifungal enzymes and detoxifies mycotoxins (AFB_1_, DON, fumonisins) or be co-applied with these microbes in an integrated biological control strategy, where plant phenolic compounds enhance that enzymatic activity [25,26]. Despite this potential, only a limited number of plant-derived extracts are commercially available. Fortunately, many plant species remain unscreened for antifungal properties, suggesting promising opportunities for discovering new molecules. Agricultural and forest byproducts represent an abundant source of raw materials with a significant potential for antifungal and antimycotoxin activities against toxigenic fungi [27,28].

Among agricultural residues, grapevine wood contains a wide variety of phenolic compounds including phenolic acids, tannins, flavonoids, and stilbenoids [29,30,31]. This large group of secondary metabolites produced via the phenylpropanoid pathways includes stilbenes which are especially abundant in the *Vitaceae* family (such as grapevines) and have been described as antifungal in bibliography [32,33]. Previous studies suggest that hydroxystilbene monomers (*trans*-resveratrol, piceatannol) and oligomers (*trans*-*ε*-viniferin, hopeaphenol, isohopeaphenol, and vitisin B) could play a role in limiting the fungal development [34,35,36]. These stilbenes are synthesized by *Vitis vinifera* in response to different stresses and can therefore be constitutively found in the canes and other woody tissues [37,38]. Traditionally, once grape leaves fall at the beginning of the dormant season, canes are pruned and either left on the soil or burned, representing an underutilized source of bioactive compounds. Therefore, with 2–5 tons of grapevine pruning residues per hectare per year, grapevine canes represent a large source of raw material in Europe and all around the world [39,40].

In the present study, we investigated the antifungal and antimycotoxigenic activity of GCE obtained via a hydro-ethanolic extraction process combined with microwave on three mycotoxigenic fungi, *F. graminearum*, *A. flavus*, and *P. expansum*. After characterization of the phenolic profile, the optimal concentration of extract required to inhibit fungal growth and supress the production of TCTB, AFLA, and PAT was evaluated. Then, we compared fungal sensitivity at early *versus* later physiological stages of development. Spores or established mycelial colonies were exposed to various concentrations of GCE, after which growth and mycotoxin production were monitored. Additionally, we tested whether the GCE acted as a germination or a mycelial inhibitor by transferring spores or colonies from treated to untreated media and checking for subsequent development. Therefore, the objectives of this work were to (1) demonstrate the extract’s biological activity, (2) elucidate the link between physiological stage and fungal sensitivity, and (3) confirm the promising potential of GCE as a natural antifungal and antimycotoxigenic agent.

## 2. Results

### 2.1. Grapevine Cane Extract as a Growth and Toxin Inhibitor of Mycotoxinogenic Fungi

**Figure 1:**
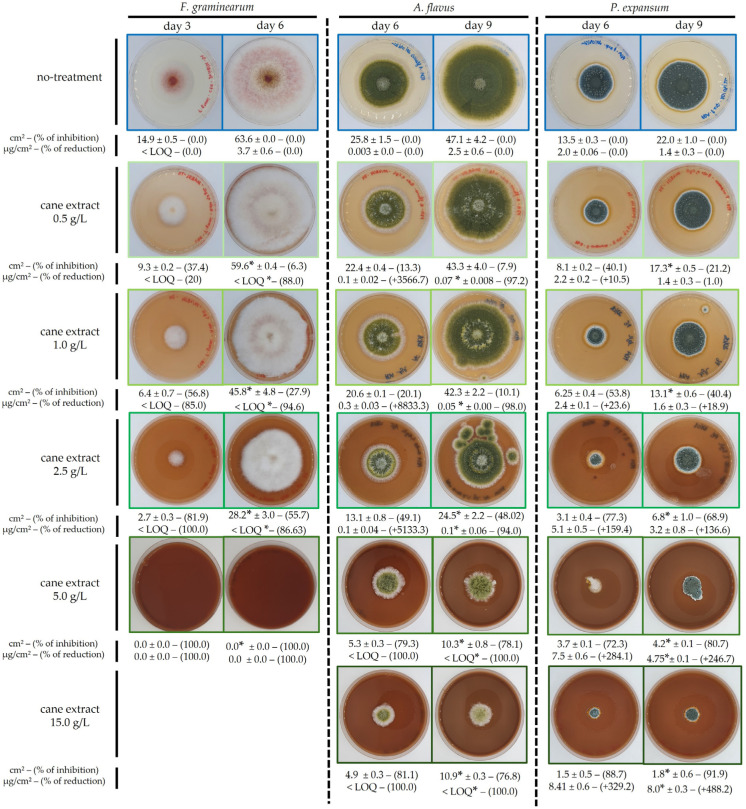
Effect of GCE on fungal growth and mycotoxin production. Fungal strains (*F. graminearum*, *A. flavus*, and *P. expansum*) were cultivated on CYA or PDA media supplemented with increasing concentrations of GCE (0.5 to 5 g/L for *F. graminearum*; and 0.5 to 15 g/L for *A. flavus* and *P. expansum*). Culture media color reflects the GCE supplementation. A commercial chemical fungicide (Imazalil 1.0 g/L) was included as a positive control; no fungal growth was observed in its presence for any of the tested species. Each column pair corresponds to a fungal species observed at two time points: *F. graminearum* (Day 3 and Day 6), *A. flavus* and *P. expansum* (Day 6 and Day 9). Below each condition, the following are mentioned: the fungal colony surface area (cm^2^), and the percentage of growth inhibition, along with the specific mycotoxin production (µg/cm^2^ of mycelium), and the percentage of reduction, relative to the untreated control. Limit of quantification (LOQ): DON = 0.9 µg/cm^2^, AFB1 = 0.001 µg/cm^2^, PAT = 0.08 × 10^−3^ µg/cm^2^. Asterisks (*) indicate conditions that differed significantly from the untreated control (*p* < 0.05; Dunn’s post-hoc test). Images are representative of three independent replicates.

The antifungal activity of GCE on fungal growth and mycotoxins production was evaluated by observing morphological changes, measuring the colony surface area, and dosing of mycotoxins in media supplemented with increasing extract concentrations (Figure 1).

For all fungi, hyphal growth and specific mycotoxin production on GCE-treated media were compared to both a negative control (no treatment) and a positive control (chemical fungicide at 1 g/L). In the presence of the chemical fungicide, fungal development and thus mycotoxins production were completely inhibited for all species and throughout the experimental period.

#### 2.1.1. Growth and Mycotoxin Inhibition by GCE (*Fusarium graminearum*)

GCE induced a significant, dose-dependent inhibition, with a visible shift from red to white observed at day 6 in *F. graminearum* (Figure 1 and Figure S1).

At 0.5 g/L and 1.0 g/L, growth was delayed, resulting in a surface area 6% and 28% smaller than the control, respectively, on day 6. At 2.5 g/L, colony expansion was substantially decreased (−55% on day 6), and full plate coverage was delayed by three days. Consistently, the 5.0 g/L treatment fully suppressed *F. graminearum* development, with no visible colony formation (Figure 1 and Figure S1). Growth rate calculations between day 3 and day 6 confirmed this pattern: 13 cm^2^/day at 1.0 g/L and 8.5 cm^2^/day at 2.5 g/L, compared to 16.0 cm^2^/day for the untreated control.

Figure 1 also presents the specific DON production (µg/cm^2^ of mycelium) by *F. graminearum*. On day 3, all conditions exhibited a DON production < LOQ = 0.9 µg/cm^2^. By day 6, DON accumulation in the untreated control increased substantially to 3.7 µg/cm^2^ of mycelium. In the presence of GCE treatments, DON was systematically below the LOQ. Table S1 indicates that 15-ADON and 3-ADON followed a similar concentration-dependent suppression pattern, confirming a broad-spectrum inhibitory effect on the trichothecene.

#### 2.1.2. Growth and Mycotoxin Inhibition by GCE (*Aspergillus flavus*)

The growth and aflatoxin B_1_ (AFB_1_) production of *A. flavus* were assessed over a 9-day incubation period. Untreated colonies displayed a characteristic green-yellowish pigmentation, reaching a surface area of approximately 47 cm^2^ and producing 2.5 µg/cm^2^ of AFB_1_ (Figure 1 and Figure S2). In GCE-treated cultures, pigmentation and colony morphology shifted in a dose-dependent manner. At 0.5 g/L, 1.0 g/L, and 2.5 g/L, colonies developed a circular aerial mycelium layer that was absent at lower concentrations, while at 5.0 g/L and 15.0 g/L, they became highly aerial and formed dome-shaped structures (Figure 1).

GCE concentrations of 0.5 g/L and 1.0 g/L had limited impact (~10% inhibition). At 2.5 g/L, 5.0 g/L, and 15.0 g/L, the colony surface areas were decreased by 48% and up to 78%. Higher concentrations (20.0 and 30.0 g/L) were tested but did not further enhance the inhibitory effect Growth rates were consistent with these measures: untreated colonies expanded at 7.1 cm^2^/day from day 6 to day 9 similar to colonies exposed to GCE at 0.5 g/L and 1.0 g/L, while rates decreased to 2.0 cm^2^/day and 1.7 cm^2^/day at 5.0 g/L and 15.0 g/L, respectively. Although GCE significantly delayed growth at its highest concentrations, complete inhibition was never observed.

Regarding mycotoxin production, on day 6, the untreated condition exhibited minimal AFB_1_ levels (0.003 µg/cm^2^), while low-to-moderate GCE concentrations (0.5 g/L–2.5 g/L) yielded higher AFB_1_ levels (0.1–0.3 µg/cm^2^), thought not significantly different from the control (Figure 1, Table S1). No AFB_1_ was detected at 5.0 g/L and 15.0 g/L, despite colony development. By day 9, while AFB_1_ accumulation in the control stabilized to 2.5 µg/cm^2^, all GCE supplemented cultures remained significantly lower (≤0.1 µg/cm^2^), with inhibition rates of ~97% (0.5 g/L), 98% (1.0 g/L), and 94% (2.5 g/L) (Figure 1). At 5.0 g/L and 15.0 g/L, AFB_1_ was not detectable. *A. flavus* predominantly synthesized AFB1, while other aflatoxins (AFB_2_, AFG_1_, AFG_2_) may have been detected at substantially lower concentrations, and when present, they exhibited a similar inhibitory response to that observed for AFB_1_ (Table S2) [41,42,43].

#### 2.1.3. Growth and Mycotoxin Inhibition by GCE (*Penicillium expansum*)

The effect of GCE on *P. expansum* was monitored over a 9-day period. Under untreated conditions, colonies reached 22 cm^2^ by day 9. Treatment with the GCE resulted in a clear, dose-dependent inhibition of growth. At 0.5 g/L and 1.0 g/L, colony surface areas were reduced to 17.5 cm^2^ and 13.5 cm^2^ (21% and 40% inhibition, respectively). Higher concentrations (2.5, 5.0, and 15.0 g/L) resulted in more pronounced reductions of 69%, 92%, and 81%, respectively (Figure 1 and Figure S3). No additional inhibition beyond 15.0 g/L was observed. Growth rate analysis (day 3–9) reflected this pattern: 3.0 cm^2^/day for both the control and the 0.5 g/L treated condition, followed by decreases to 2.3, 1.2, 0.1, 0.9 cm^2^/day at 1.0, 2.5, 5.0, and 15.0 g/L, respectively. Overall, GCE induced between 21% and 91% growth inhibition, although complete growth suppression was not achieved under the tested conditions.

Patulin production was also influenced by GCE, as shown in Figure 1 and Table S1. At day 6, control cultures produced 2.0 µg/cm^2^ of patulin. Lower extract concentrations (0.5–1.0 g L) yielded similar or slightly elevated values (2.2, 2.4 µg/cm; +10% to +19%). However, higher concentrations (2.5, 5.0, and 15.0 g/L) resulted in significantly increased patulin levels from 159% to 329%. By day 9, patulin levels in the control stabilized to 1.4 µg/cm^2^. Lower GCE treatments (0.5 g/L, 1.0 g/L) kept specific patulin production relatively close to the control (1.4 and 1.6 µg/cm^2^), while higher concentrations (2.5, 5.0, 15.0 g/L) led to significant increases, from 237% to 587% (Figure 1, Table S1).

### 2.2. Inhibition Through Fungal Life Cycle: From Spores to Expanding Mycelium

**Figure 2:**
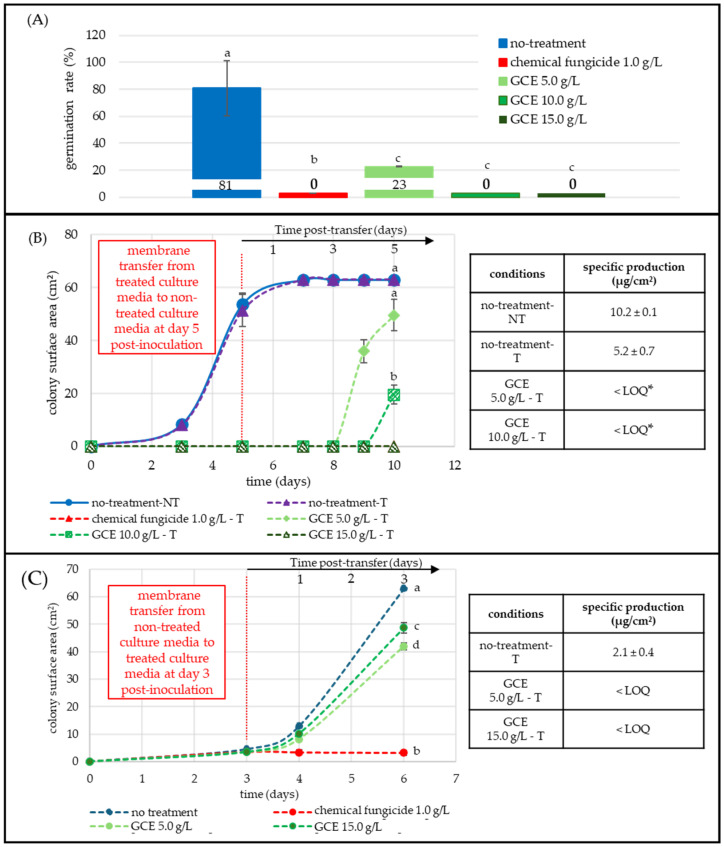
Effect of GCE on *F. graminearum* germination, growth, and DON-specific production (µg/cm^2^ of mycelium) at different developmental stages. All GCE treatments 5.0, 10.0, 15.0 g/L (green shades) were compared to non-treated (blue) and fungicide-treated (1 g/L, red) conditions. Each point represents the mean of three replicates. DON LOQ = 0.9 µg/cm^2^. (**A**) Spore germination assay: spores were inoculated on membranes placed over treated CYA medium; germination was evaluated microscopically (20×–40×) 12 h post-inoculation, (50 spores/image, three replicates). A spore was considered germinated when the germ tube exceeded twice the spore length. (**B**) Post-transfer assay: spores were first grown on GCE supplemented media, then transferred (T) to untreated media on day 5 (vertical red dashed line). Colony growth (cm^2^) and DON-specific production (µg/cm^2^) were monitored until day 10. (**C**) Mycelial-stage assay: Colonies were grown for 3 days on untreated media, then transferred to GCE-supplemented media. Growth and DON-specific production were measured until day 6. Statistical significance (*p* < 0.05) was assessed using the Kruskal-Wallis test, followed by Dunn’s post-hoc test. Different letters indicate significant differences in germination and growth; asterisks indicate differences in DON production. Curves at 0 are superimposed; the red series is present but coincides with other zero-valued conditions and is therefore not visible.

**Figure 3:**
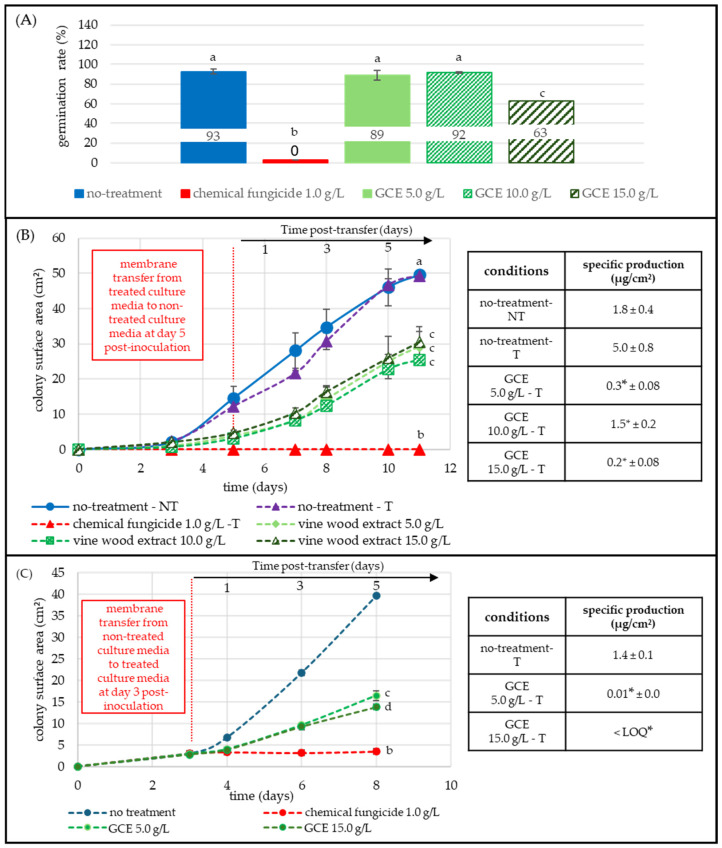
Effect of GCE on *A. flavus* germination, growth, and AFB_1_ production at different developmental stages. All GCE treatments (5.0, 10.0, 15.0 g/L; green shades) were compared to non-treated (blue) and fungicide-treated (1 g/L, red) conditions. Each point represents the mean of three replicates. AFB_1_ LOQ = 0.001 µg/cm^2^. Panel (**A**) Spore germination assay: spores were inoculated on membranes over GCE-treated PDA medium; germination was assessed microscopically (20×–40×) 12 h post-inoculation (50 spores/image, three replicates). Panel (**B**) Post-transfer assay: spores were pre-grown on extract-supplemented media and transferred (T) to untreated media on day 5 (vertical red dashed line). Colony growth (cm^2^) and AFB_1_ specific production (µg/cm^2^) were monitored until day 11. Panel (**C**) Mycelial-stage assay: colonies pre-formed for 3 days on untreated medium were transferred to GCE-supplemented media. Growth and AFB_1_ specific production were evaluated until day 8. Statistical significance (*p* < 0.05) was determined using the Kruskal-Wallis test, followed by Dunn’s post-hoc test. Different letters indicate significant differences in germination and growth; asterisks indicate differences in specific AFB_1_ production.

**Figure 4:**
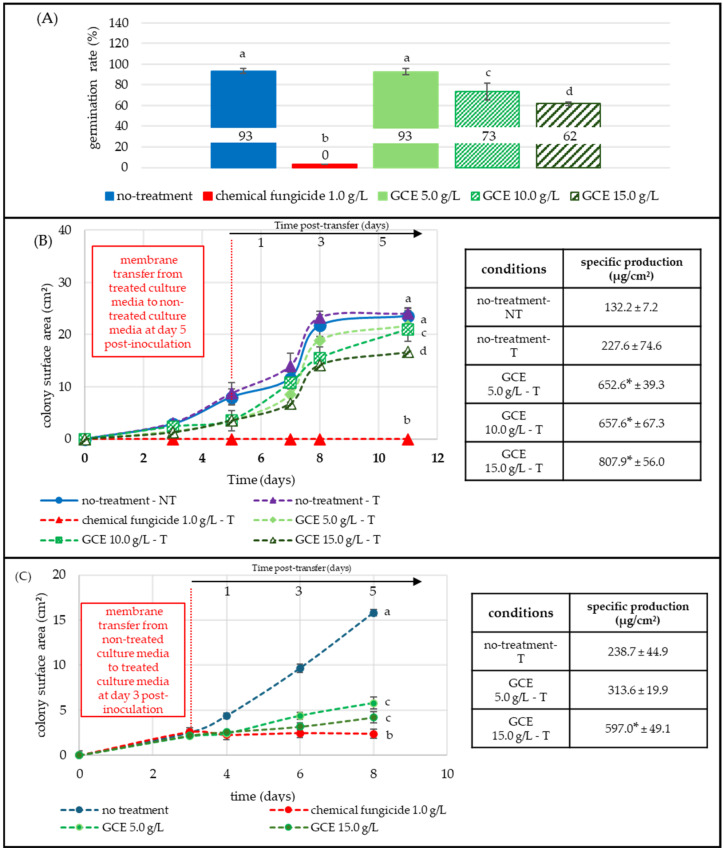
Effect of GCE on *P. expansum* germination, growth, and PAT production at different developmental stages. All GCE treatments 5.0, 10.0, 15.0 g/L (green shades) were compared to non-treated (blue) and fungicide-treated (1 g/L, red) conditions. Each point represents the mean of three replicates. PAT LOQ = 0.08 × 10^−3^ µg/cm^2^. Panel (**A**) Spore germination assay: spores were inoculated on membranes placed over treated PDA medium; germination was assessed microscopically (20×–40×) 12 h post-inoculation (50 spores/image, three replicates). Panel (**B**) Post-transfer assay: spores were first grown on extract-supplemented media and transferred (T) to untreated media on day 5 (vertical red dashed line). Colony growth (cm^2^) and PAT specific production (µg/cm^2^) were monitored until day 11. Panel (**C**) Mycelial-stage assay: 3-day-old colonies grown on untreated culture media were transferred to GCE-supplemented media; colony surface and patulin specific production were evaluated up to day 8. Statistical significance (*p* < 0.05) was determined using the Kruskal-Wallis test, followed by Dunn’s post-hoc test. Significant differences in germination and growth are shown with letters; significance in patulin specific production is indicated with an asterisk.

To assess whether GCE impacts fungal development and mycotoxin production at different life-cycle stages, three complementary approaches were employed. (1) First, spores (macroconidia—*F. graminearum*, or conidia–*A. flavus* and *P. expansum*) were inoculated on porous membranes placed over culture media supplemented, or not, with GCE at various concentrations, to determine whether germination was blocked or delayed (Figure 2, Figure 3 and Figure 4A). (2) Next, to determine whether early exposure to GCE exerted a lasting effect on fungal growth and toxin production, or if normal development resumed once GCE was removed, germinated or non-germinated spores were transferred from treated to untreated media (Figure 2, Figure 3 and Figure 4B). (3) Finally, the capacity of GCE to inhibit active mycelial growth and ongoing toxin production, and thus exert a potential curative effect, was evaluated. Spores were first germinated and microscopic colonies were allowed to develop on untreated media before being transferred to GCE-supplemented plates (Figure 2, Figure 3 and Figure 4C). Together, these approaches provided insight into whether the antifungal and antimycotoxigenic activities of GCE were limited to early development stages or extended to mature, actively growing colonies.

#### 2.2.1. *Fusarium graminearum*: An Early-Stage Sensitive Fungi: Germination Inhibition and Remanence of Antifungal Effect

Figure 2A showed that a 12 h exposure at 25 °C to GCE (10.0 and 15.0 g/L) completely blocked the germ-tube emergence and reduced the germination rate from 81% (untreated control) to 23% at 5.0 g/L. The commercial fungicide (1.0 g/L) also completely inhibited germination.

Figure 2B quantified both growth recovery and toxin output following early exposure to GCE and subsequent transfer to fresh CYA medium. Spores were first incubated for 5 days on media containing 5.0, 10.0, and 15.0 g/L of GCE, after which the membranes were transferred to fresh non-supplemented plates (dashed vertical red line). Non-transferred (NT-blue) and transferred (T-purple, dashed) controls displayed identical growth curves, confirming that transfer did not limit expansion (≈53.0 cm^2^ on day 5 and 63.0 cm^2^ on day 10, respectively). Colonies exposed to 5.0 and 10.0 g/L did not develop before transfer, but resumed growth afterward, reaching 49.6 cm^2^ and 20.0 cm^2^ by day 10, which represents a 21% and 68% reduction in surface area, respectively (Figure 2). The 15.0 g/L treatment fully blocked fungal development. No development was noted until 15 days.

Specific DON production corroborated these profiles: the untreated transferred control (T-purple, dashed) produced 5.2 ug/cm^2^, while early exposure to 5.0 g/L GCE reduced DON-specific production to 0.4 g/L (−92%), and 10.0 g/L kept DON below LOQ (Figure 2); 15-ADON followed the same pattern as DON, whereas 3-ADON stayed < LOQ under all conditions tested including controls (Table S3). Because the graph presents both, absolute and post-transfer timelines, colonies of equivalent physiological age can be compared. Control conditions (blue, purple) plotted on the absolute timeline had already reached their maximal diameter by the time the treated colonies began to expand. Thus, a five-day post-transferred colony (dashed—green shades) that had been inhibited by a 5.0 or 10.0 g/L GCE corresponded to an absolute age of ten days but still exhibited a five-day-old control equivalent (−3%) or smaller (−61%) surface area, respectively. DON levels from five-day post-transfer colonies were far lower (−85% inhibition) than the five-day-old control (≈50.0 cm^2^, 2.7 µg/cm^2^). Toxin production in treated culture did not catch up with untreated controls during the experiment.

Figure 2C addressed later physiological stages. Three-day-old colonies (≈3.0 cm^2^ in diameter), grown on non-treated medium until they reached a mycelial stage, were transferred to plates containing 5.0 or 15.0 g/L GCE, or to Imazalil-supplemented medium (1.0 g/L). The untreated, transferred control expanded rapidly to 63 cm^2^ by day 6, while colonies exposed to the fungicide control barely progressed (≈3 cm^2^). The extract slowed radial growth but did not fully stop it, with average final areas of 41.9 cm^2^ at 5.0 g/L (−42% vs. control) and 48.7 cm^2^ at 15.0 g/L (−23% vs. control) (Figure 2).

DON levels on day 6 revealed a stronger contrast between non-treated (blue) and treated (green shades) conditions. The untreated control accumulated 2.1 µg/cm^2^, while both extract treatments kept DON below LOQ. The same observation was made for 15-ADON and 3-ADON (Table S4).

#### 2.2.2. *Aspergillus flavus*: Moderate Germination Inhibition with Sustained Mycelial Suppression and Remanence of Antifungal Effect

Figure 3A presents the impact of GCE on spore germination after 12 h. Under control conditions (no treatment, blue) 93% of spores germinated, while no germination was observed when treated with the chemical fungicide (1 g/L, red). GCE at 5.0 g/L and 10.0 g/L had minimal impact on germination, with rates of 89% and 92%, respectively. The 15 g/L concentration reduced the germination rate to 63%, only the highest dose significantly interfered with the germination of *A. flavus* spores.

Figure 3A tracked colony establishment following germination. *A. flavus* spores were incubated for 5 days on GCE-supplemented medium, after which the membranes were transferred to fresh, non-treated PDA (vertical dashed red line). Non-transferred (NT-blue) and transferred (T-purple, dashed) controls grew identically, both reaching 49.3 cm^2^ by day 11, indicating that the transfer itself did not restrict the growth. Colonies pre-exposed to 5.0, 10.0, and 15.0 g/L of GCE were partially inhibited, achieving final areas of 29.3 cm^2^ (−40% vs. control), 25.5 cm^2^ (−48% vs. control), and 30.5 cm^2^ (−38% vs. control), respectively (Figure 3). The three GCE treatments (5.0, 10.0, and 15.0 g/L) inhibited colony expansion to a similar extent, and their final surface areas did not differ significantly from one another. No fungal development was observed in the presence of the fungicide treatment.

Mycotoxin analysis revealed that early extract exposure reduced AFB_1_ accumulation. The transferred control (T-purple, dashed) produced 5.0 µg/cm^2^ of AFB_1_ by day 11, while colonies exposed to 5.0 and 15.0 g/L accumulated only 0.3 and 0.2 µg/cm^2^, respectively, corresponding to 94% and 96% reductions (Figure 3, Table S5). The 10 g/L concentration achieved a 70% reduction.

Figure 3C evaluated the response of *A. flavus* once colonies had reached the mycelial stage. Three-day-old colonies (≈3 cm^2^) grown on non-supplemented PDA were transferred (vertical dashed red line) to plates containing 5.0 or 15.0 g/L GCE, or to fungicide-supplemented media (1 g/L, dashed red). The untreated, transferred control (T, dashed blue) developed rapidly after transfer, with its area increasing from 2.8 cm^2^ to 39.7 cm^2^ within five days. Exposure to GCE slowed growth, resulting in final surface areas of 16.5 cm^2^ (−58%) at 5.0 g/L and 13.8 cm^2^ (−65%) at 15.0 g/L. The fungicide fully stopped the expansion of *A. flavus* colonies.

AFB1 quantification on day 8 revealed a pronounced inhibition effect. The transferred control (T, dashed blue) produced 1.4 µg/cm^2^, whereas colonies exposed to 5.0 g/L accumulated only 0.01 µg/cm^2^ (−99%), and 15.0 g/L kept AFB1 < LOQ (Figure 3, Table S6).

#### 2.2.3. *Penicillium expansum*: Moderate Germination Inhibition with Strong and Persistent Mycelial Suppression

Figure 4A illustrates the *P. expansum* germination rate in the presence of GCE after 12 h of incubation at 25 °C. Under control condition, 93% of spores germinated, while the chemical fungicide (1 g/L, red) condition showed no germination. GCE at 5.0 g/L did not significantly affect the germination rate, while at 10.0 g/L and 15.0 g/L, germination decreased to 73% and 62%, respectively.

Figure 4B followed colony development after five-day exposure to 5.0, 10.0, and 15.0 g/L GCE and subsequent transfer (vertical dashed red line) to untreated, fresh PDA medium. Colony growth and mycotoxin production were monitored until day 11. Non-transferred (NT-blue) and transferred (T-purple, dashed) controls expanded identically, both reaching ≈ 24 cm^2^ by day 11, confirming that the transfer step itself did not restrict growth. Pre-exposure to 5.0, 10.0, and 15.0 g/L GCE reduced final colony size to 21.7 cm^2^ (−10%), 21.0 cm ^2^ (−12%), and 16.6 cm^2^ (−31%), respectively (Figure 4).

In contrast, PAT-specific production increased in the presence of GCE. The transferred control (T-purple, dashed) produced 227.6 µg/cm^2^ of PAT, while the specific production in colonies initially exposed to GCE increased to 652.6 µg/cm^2^ (+65%) at 5.0 g/L, 657.6 µg/cm^2^ (+65%) at 10.0 g/L, and 807.9 µg/cm^2^ (+72%) at 15.0 g/L (Figure 4, Table S7).

Figure 4C assessed *P. expansum* growth and PAT production from colonies that had already reached a mycelial stage. Three-day-old colonies (≈2.5 cm^2^) grown on non-treated media were transferred (vertical dashed red line) to GCE-supplemented media, or to fungicide-supplemented medium. The untreated transferred control (T-purple, dashed) expanded to 15.8 cm^2^ by day 8, whereas 5.0 g/L and 15.0 g/L limited fungal growth to 5.8 cm^2^ (−63%) and 4.2 cm^2^ (73%), respectively. The fungicide blocked the fungal development.

Specific PAT production of the non-treated control reached 238.7 µg/cm^2^ after 8 days. In the presence of GCE, PAT-specific production increased to 313.6 µg/cm^2^ (+24%) at 5.0 g/L and 597.0 (+60%) at 15.0 g/L (Figure 4, Table S8).

## 3. Discussion

The current study shows that GCE, obtained by the hydro-ethanolic extraction process combined with microwaves treatment, affected both growth and mycotoxin production in all three targeted fungi, *F. graminearum* (BRFM 1967 strain), *A. flavus* (NRRL62477, E73 strain), and *P. expansum* (NRRL 35695 strain), but displayed species-specific patterns. We did not perform viability assays or determine MIC/MFC; results are therefore reported as endpoint-specific inhibition under the tested conditions, without any claim of lethality. Radial growth IC_50_ values clustered between 1.0 and 5.0 g/L for all models (Figure 1; Table 1). While GCE exhibited a pronounced inhibitory effect against *F. graminearum*, fully stopping growth at concentrations ranging from 2.5 g/L to 5.0 g/L, it also showed complete and remanent inhibition of germination at 15.0 g/L (Figure 2B; Table 1). No total inhibition of germination was observed for *A. flavus* and *P. expansum*, and mycelial growth was not abolished for any species under the tested conditions at the mycelial state (Figure 1; Table 1). Complete growth inhibition was not achieved, even at 30.0 g/L; however, a concentration of 15.0 g/L still suppressed growth by approximately 78% and 92%, for *P. expansum* and *A. flavus*, respectively, demonstrating a strong inhibitory effect. These concentrations were of the same order of magnitude as those reported for other aqueous plant extracts. For example, an aqueous maritime-pine sawdust extract and GCE suppressed *F. graminearum* growth by 60–89% at 3.7 g/L and by 50% at 11.3 g/L, respectively [27]. Other studies in the bibliography confirmed that similarly high extract doses are needed to inhibit these fungi: 7.5 g/L of *Adenocalymma alliaceum* extract reduced *A. flavus* growth by 51%, and 30.0 g/L of *Piper nigrum* extract suppressed *P. expansum* growth by 60% [44,45].

In addition to its antifungal effects, GCE significantly impacted mycotoxin production in all three fungi studied. DON and its acetylated derivatives produced by *F. graminearum* were strongly suppressed in a dose-dependent manner. At subinhibitory concentration (0.5 g/L), DON production decreased by 88% and dropped below 1 µg/cm^2^ at 1 g/L, even though mycelial growth persisted up to 5 g/L (Figure 1; Table 1). This result aligns with previous studies reporting TCTB inhibition by polyphenol-rich extracts, like grapevine derivatives (Table 2) [28,29,30,31,32,33,34,35,36,37,38,39,40,41,42,43,44,45,46]. Similarly, AFB_1_ levels in *A. flavus* dropped by ≥95% at doses that reduced radial growth by only 10% (Figure 1; Table 1). On day 6, low-to-moderate extract concentrations (0.5–2.5 g/L) transiently increased AFB_1_ levels, reaching a maximum at 1.0 g/L. This stimulatory effect may reflect stress-induced overactivation of the aflatoxin biosynthetic pathway. In contrast, the response of *P. expansum* to GCE differed markedly with a dose-dependent increase in patulin production, reaching a six-fold rise at 5.0 g/L despite a 91% inhibition of fungal growth (Figure 1; Table 1). This enhancement may also result from stress-induced activation of toxin biosynthesis. Across species, GCE inhibited mycotoxin production at lower concentrations than those required to inhibit growth, revealing a complex and species-specific dose-response pattern (Figure 1; Table 1).

*F. graminearum* was the most impacted fungus during the germination stage. In Figure 2A, the extract inhibited spore germination by 72–100% at moderate (5.0 g/L) and high (15.0 g/L) concentrations, suggesting interference with one or more early morphogenetic steps. In contrast, germination was only moderately inhibited at the highest concentrations of GCE for *A. flavus* and *P. expansum*, with 32% and 33% lower germination rate, respectively (Figure 3A and Figure 4A). These results were consistent with reports showing a global but species-dependent anti-germinative activity of polyphenols [28,29,30,31,32,33,34,35,36,37,38,39,40,41,42,43,44,45,46,47,48,49].

After the spores were transferred from treated to fresh, untreated medium (Figure 2B and Figure 3B), the expansion of *F. graminearum* and *A. flavus* colonies was either delayed or slowed down, while specific DON and AFB_1_ levels dropped by 92–100%. *F. graminearum* colonies previously exposed to 5.0 g/L GCE eventually recovered, reaching a surface area comparable to the 5-day, non-treated control. This confirms a concentration-dependent yet persistent antifungal effect, extending beyond direct contact between the fungus and GCE. The sustained reduction in both biomass, DON and AFB_1_, indicates a measurable remanent effect. At 15.0 g/L, no spore germination was observed, and *F. graminearum* spores did not recover after transfer to GCE-free medium over five days, indicating a strong, sustained inhibitory effect under the tested conditions. Further experiments are mandatory to further confirm any potential sporicidal effects. *A. flavus* growth inhibition was maximal at 5.0 g/L, higher doses (10.0 and 15.0 g/L) produced no additional inhibition, suggesting the maximal inhibitory threshold had already been reached. *P. expansum* responded differently. In Figure 4B, patulin production increased, increasing from 5.0 to 15.0 g/L despite progressive growth suppression, suggesting that the extract may have induced metabolic stress that triggered the patulin biosynthetic pathway. Growth resumed slowly after transfer to non-treated medium, yet patulin levels remained elevated, underscoring a dissociation between radial growth and toxin output.

When the GCE extract was applied to pre-grown mycelia, the impact on growth was more limited (Figure 2C, Figure 3C and Figure 4C). *F. graminearum,* which was sensitive during germination, became comparatively resilient once a mycelial colony was established, exhibiting only 22% growth inhibition, while *A. flavus* showed the opposite pattern: little inhibition during germination but a 62% reduction at the mycelial stage acting mainly as a growth inhibitor. DON and AFB_1_ remained undetectable at 5.0 and 15.0 g/L, respectively, showing that the extract permanently inhibits TCTB and AFLA biosynthesis even without complete growth inhibition. These results confirmed the extract’s ability to disrupt secondary metabolism even in mature colonies.

In Figure 4C, patulin continued to increase even when *P. expansum* colony development was limited, highlighting a clear decoupling of growth and toxin-specific production. Although *P. expansum* was more resistant than *F. graminearum* at the germination stage, it proved to be the most sensitive species once mycelia had formed, displaying a 69% inhibition of radial growth at 15.0 g/L.

Taken together, these stage-specific experiments emphasized both fungal-specific differences and common patterns. *F. graminearum* exhibited a tightly linked decline in growth and DON production, with DON dropping below the limit of quantification at doses that still allowed for some radial expansion. *A. flavus* responded more complexly: moderate GCE concentrations partially slowed growth, whereas higher concentrations completely suppressed AFB_1_. In contrast, *P. expansum* showed the opposite pattern, patulin levels rose sharply under extract-induced stress even as growth was strongly inhibited. Despite species-specific responses, two similarities emerged. First, intermediate extract doses completely blocked toxin formation in both *F. graminearum* and *A. flavus*. Second, growth and toxin suppression persisted after the extract was removed, showing that the effect was not fully reversible. The pronounced uncoupling of radial growth and toxin inhibition in these fungi suggests that GCE might primarily target secondary metabolism regulation, likely by down-regulating key genes such as *TRI5* (*F. graminearum*) and *aflR* (*A. flavus*), rather than merely limiting biomass [50,51]. *P. expansum*, in contrast, strongly increased patulin accumulation when stressed by GCE, indicating an alternative stress-response mechanism that activates toxin biosynthesis, consistent with reports of patulin upregulation under sub-lethal oxidative stress [52].

The GCE used in this study was obtained from grapevine cane (*Vitis vinifera*). The biochemical composition of GCE has been described in detail over many years, consistently showing a high content of phenolic compounds, including stilbenes [53,54,55,56]. *Trans*-resveratrol, the best-known stilbene and precursor of many others, is the predominant monomer in this extract [32,33] (Table S9). In plants, *trans*-resveratrol and piceatannol can be further oligomerized, glycolyzed, methylated, isomerized, or isoprenylated, generating a wide spectrum of derivatives [56]. The same molecular diversity was evident in our extract (Table S9). Our microwave-assisted hydro-ethanolic extract contained 210 g of stilbenes per kg of GCE powder. This high proportion reflects the extraction process: microwaves hydro-ethanolic extraction followed by evaporation, concentration, and drying concentrates of the stilbenes in the final powder extract. Because composition influences bioactivity, the main constituents of the extract were summarized in Table 2 and Table S9.

Stilbenes are well-known phytoalexins that accumulate in grapevine tissues during fungal attack [38,39,40,41,42,43,44,45,46,47,48,49,50,51,52,53]. Pezet et al. (2003) demonstrated that their accumulation increases in lignified tissues infected by *Plasmopara viticola*, the causal agent of downy mildew [57]. This defensive response has since been confirmed in several studies, including those involving other ascomycete pathogens [34,35,36,37,38] (Table 3). In addition, stilbenes identified in our extract, such as *trans*-resveratrol, piceatannol, *trans*-*ε*-viniferin, and vitisin B, have antifungal and antimycotoxigenic activities that matched those reported for purified compounds at an equivalent concentration (Table 3). For example, *trans*-resveratrol, *trans*-*ε*-viniferin, and piceatannol inhibit *Plasmopora viticola* and *Botrytis cinierea* at ≈60–120 µM and ≈60–400 µM, respectively, while our extract contains 72.4–233.4 µM of resveratrol and 6.4–39.7 µM of piceatannol in the active concentration range from 1 to 5 g/L [34,35,36].

Tardif et al. (2025) compared cane, wood, and root grapevine extracts from several grapevine cultivars against *F. graminearum*; all fractions suppressed growth and TCTB production in a dose- and cultivar-dependent manner, although cane extracts were the least active [50]. In our study, the concentrations of *trans*-resveratrol, *trans*-*ε*-viniferin, and piceatannol delivered by a 2.5 g/L to 5.0 g/L cane extract matched those concentrations and abolished TCTB production while strongly inhibiting fungal growth. In *A. flavus*, *trans*-resveratrol and related phenolics down-regulate aflatoxin-cluster genes, yet this inhibition requires higher doses than those effective against *F. graminearum*. Similarly, stronger concentrations are needed to slow *A. flavus* mycelial growth, a difference that might be explained by its thicker, more hydrophobic conidial wall [58,59].

Consistent with these findings, the highest doses of GCE lowered AFB_1_ by 80% and strongly inhibited colony expansion. In contrast, *P. expansum* was only moderately inhibited, showing limited growth reduction and a dose-dependent increase in patulin, as previously reported [52]. Across all three fungi, toxin suppression occurred at extract doses below those required for substantial growth inhibition, a pattern documented for polyphenols [60,61,62]. Therefore, stilbenoids might primarily target the secondary-metabolite pathway and gene clusters (e.g., *TRI5*, *aflR*) rather than impacting cellular and metabolic processes involved in biomass accumulation.

Due to methodological differences (strain origin, extraction technique, culture conditions) cross-study comparisons (Table 2) are limited. While prior work often focused on single phenolic molecules (e.g., *trans*-resveratrol), the GCE is a mixture. Not all phenolic molecules mentioned in those studies were stilbenoids (e.g., non-stilbene classes like caffeic acid and quercetin). Such variability limits direct quantitative comparisons, yet data obtained with GCE and presented in this article were consistent with prior results: phenolics compounds alone or in a mixture (plant extract) can lower mycotoxin accumulation even when growth effects are modest; growth suppression did not always mirror toxin reduction; both GCE and single phenolic compound or other grapevine extracts appear to have consistent inhibitory action.

Stilbenes such as ampelopsin A, hopeaphenol, *trans*-resveratrol, *trans*-*ε*-viniferin, and *trans*-vitisin B were reported to disorganize the plasma membrane and organelle membranes of *P. viticola* and *B. cinerea* spores, an effect confirmed in several follow-up studies [49,50,51,52,53,54,55,56,57,58,59,60,61,62,63,64]. Phan et al. (2014) further demonstrated that, in fungi, flavonoids tend to rigidify membranes, whereas stilbenes increase fluidity [65]. Given the high content of *trans-ε*-viniferin (a resveratrol dimer) in our extract, and the greater antifungal efficacy reported for resveratrol oligomers, as their degree of polymerization and log P increases, disrupting the fungal cell wall and plasma membrane which control nutrient uptake, defence and permeability might be one of the underlying mechanisms of action of GCE [66,67].

Once inside the cell, phenolic compounds can disrupt both primary and secondary metabolism. For example, *trans*-resveratrol at much lower concentrations than those present in our extract down-regulated *aflaA* and *aflaB* involved in aflatoxin synthesis in *A. flavus* and simultaneously inhibited mycelial development and sporulation [51]. These reports supported our observation that toxin repression occurred at doses an order of magnitude below those required for strong growth inhibition. Finally, free diffusion of phenolic compounds inside the cell can disturb oxidative-stress regulation and mitochondrial function, altering the cellular ROS balance [68,69]. Although direct data on stilbene-induced metabolic and mitochondrial dysfunction remain limited, the combined evidence of gene down-regulation, ROS scavenging and ETC inhibition for related phenolics compounds supports a similar mode of action for stilbenes present in GCE [70,71]. Studies have shown that antioxidant phenolics compounds scavenge ROS and supress aflatoxin and trichothecene biosynthesis in *A. flavus* and *F. graminearum* [72,73]. Hydrogen bonding between phenolic hydroxyls and enzymes may amplify this disruption.

The extract shows promising in vitro activity, but turning GCE into a robust phytopharmaceutical product (P.P.P) will depend, in part, on its effectiveness on fields and on future formulation strategies. The efficacy of the extract has been tested and demonstrated on fields for apple-scab (*Venturia inaequalis*), which has been chosen as the representative use for the registration as P.P.P, following E.U. regulation 1107/2009 [74]. In this study, GCE was used as a powder, but liquid formulations are more suitable for field use. Indeed, powder extracts are no longer of interest due to higher risk of exposure for farmers and poor water solubility of active molecules such as phenolic compounds. Formulation approaches adapted from the cosmetics and pharmaceutical sectors can enhance solubility, stability, and prevent rainfastness [75]. Indeed, the amphiphilic nature of phenolic compounds such as stilbenes, governs adsorption, aggregation, complexation, and micellization, thereby shaping antifungal efficacy. Combining plant fractions that act on complementary cellular targets, then encapsulating and formulating them, should protect the actives, concentrate them on plant surfaces, and deliver adequate doses to their sites of action. Practical solutions include nanoemulsions (including Pickering systems), nanocrystals, phytosomes, or lipid nanoparticles [76,77,78,79]. For field applications, water-based sprays are preferred, but hydrophobic leaf surfaces promote runoff, incorporating carriers into aqueous solutions that turn into hydrogels (e.g., tannin- or polysaccharide-based can improve adhesion and rainfastness).

## 4. Conclusions

This study demonstrated that a stilbenoid-rich extract from grapevine cane, obtained through an industrial extraction process combining microwaves, ultrasound, and vacuum is a potential antifungal and antimycotoxigenic agent. In vitro Petri dish culture showed that low concentrations (as low as 0.5 g/L) suppressed TCTB and AFLA production by approximately 90%, while growth IC_50_ values ranged from 1.0 to 5.0 g/L. Although patulin production increased in the presence of GCE, its hyphal development was partially inhibited. These results might indicate that growth inhibition and mycotoxin suppression are uncoupled phenomena, each likely driven by distinct modes of action. Stage-specific assays revealed that spores were more vulnerable than established mycelia for *F. graminearum*, and that the extract retained partial activity even after removal, confirming true inhibitor remanence. Comparison with IC_50_ values from the bibliography indicated that the concentrations of *trans*-resveratrol, piceatannol, *trans*-*ε*-viniferin and vitisin B delivered by GCE were comparable to a certain extent. The results indicate that higher GCE concentrations are associated with both stronger antifungal and antimycotoxigenic effects, plausibly reflecting the concentration and molecular structures of its stilbenes, with potential synergistic, antagonistic, or agonistic interactions among components. Moreover, previous studies have identified structural and functional fungal targets that may explain species-dependent effects. To further elucidate its mechanisms of action, comparative studies on extract fractions and purified molecules are needed to determine the specific role of stilbenes in its antifungal and antimycotoxin activity. Moreover, the mode of action should be explored through the study of GCE impact on cellular oxidative state after exposure.

## 5. Materials and Methods

### 5.1. Grapevine Cane Extract (GCE)

**Figure 5:**
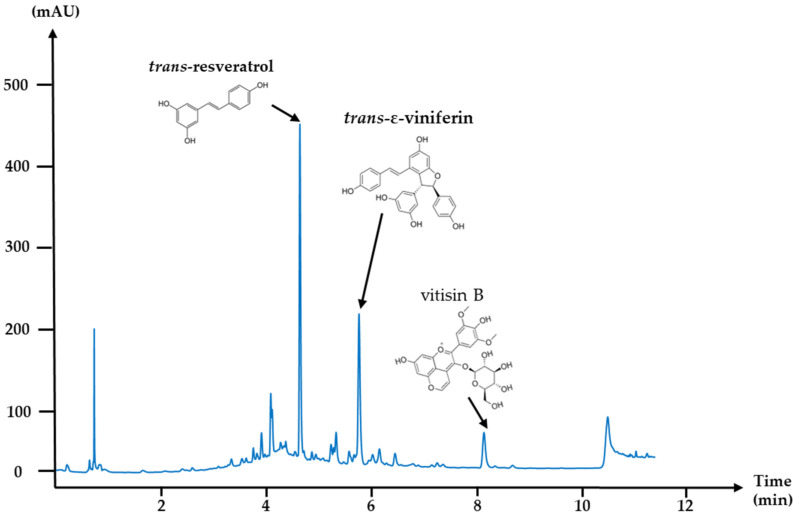
UHPLC-DAD chromatogram at 254 nm of the grapevine cane extract (*V. vinifera*) at 5 g/L solubilized in ethanol 50% (*v*/*v*). The analysis was set using a C18 column in reverse phase mode, the elution was performed at a flow rate of 1.6 mL/min with a volume of injection of 5 µL.

One-year old grapevine canes from *Vitis vinifera*, variety “Pinot noir”, were collected on the pruning season (2021) from several regions in France. Grapevine canes were cut into small segments of 10 to 20 cm, then crushed into smaller fragments (2 to 10 mm) using a conventional knives grinder equipped with sieves. The ground grapevine canes were washed for 1 h in hot water (90 °C) to clean the raw material and remove most of the sugars present in the wood; finally, the cleaning water was eliminated. The ground, humidified wood material was mixed with 30% (*v*/*v*) aqueous ethanol. Microwaves (1–100 kW) combined with ultrasound and vacuum, as described in Antofénol patent from 2020, were applied to the grapevine cane/EtOH/water mixture for 1 h, over 80 °C. The resulting liquid extract was filtered on a 20 μm filter, evaporated and spray-dried into a fine brown powder, which was packaged and stored in the dark at room temperature until further use. At the end of this process, a control was performed to check the biochemical composition of the extract by HPLC with a UV detector at 254 nm, as detailed in Table 4: HPLC mobile phase gradient and Figure 5: UHPLC-DAD chromatogram at 254 nm of the grapevine cane extract (*V. vinifera*) at 5 g/L solubilized in ethanol 50% (*v*/*v*). A single industrial eco-extract of Pinot Noir grapevine cane with a very reproducible phenolic composition was used for the entire experiment.

The grapevine cane extract was produced in batches, with a specific batch used for all experiments in this study. Following production, its biochemical profile was analyzed using UHPLC (Vanquish CORE, Thermo Fisher, Villebon-sur-Yvette, France) coupled with a UV-Vis DAD detector. The separation was performed on a C18 Accucore RP-MS column (100 × 4.6 mm, 2.6 µm, ThermoScientific).

The mobile phase consisted of 0.1% formic acid (*v*/*v*) (Sigma-Aldrich, Saint-Quentin-Fallavier, France, F0507-1L, CAS: 64-18-6) in water (solvent A) and 0.1% formic acid (*v*/*v*) in acetonitrile (FisherBioScientificVillebon-sur-Yvette, France, HPLC gradient grade, CAS: 75-05-8) (solvent B). A 5 g/L solution of the extract was prepared in 50% ethanol (*v*/*v*) to enhance the solubility of hydrophobic compounds, particularly stilbene oligomers. Analysis was performed at 254 nm to optimize stilbene detection. Three key compounds, *trans*-resveratrol, *trans*-*ε*-viniferin, and vitisin B, were specifically monitored as biomarkers of the extract.

### 5.2. Microorganisms, Storage Conditions, and Fungal Culture

#### 5.2.1. *Fusarium graminearum*

*F. graminearum* strain CIRM-BRFM 1967 (CIRM, University of Aix-Marseille, Marseille, France) was selected for its documented high mycotoxin production; the same culture-collection strain (BRFM 1967) was used in [80,81]. On CYA and PDA (25 °C, 7 days), colonies grow rapidly forming a pale to red abundant aerial mycelium. Numerous macroconidia (large asexual spores) are produced: curved, multiseptated. Originally isolated from a wheat plant, it exhibits a deoxynivalenol (DON), 15-acetyldeoxynivalenol (15-ADON), and 3-acetyldeoxynivalenol (3-ADON) chemotype. The strain was maintained on potato dextrose agar (PDA; Biokar diagnostics, Beauvais, France) under paraffin oil at 4 °C. For spore production, the strain was inoculated on PDA agar and grown for 4 days. Mycelium plugs were then transferred to carboxymethyl cellulose liquid media (CMC) and incubated for 96 h in the dark, with agitation at 150 rpm, 25 °C. The CMC medium was prepared by dissolving the following components in 1 L of demineralized water: 0.5 g of magnesium sulfate heptahydrate (MgSO_4_.7H_2_O, Fluka honywell, Sigma Aldrish, Saint-Quentin-Fallavier, France, CAS: 10034-99-8), 1 g of ammonium nitrate (NH_4_NO_3_, Sigma-Aldrich, CAS: 6484-52-2), 1 g of potassium dihydrogen phosphate (Sigma-Aldrich, CAS: 7778-77-0), 1 g of yeast extract (powder) (BioKar diagnostics, Allonne, France, reference A1202 HA, 500 g), and 1 g of carboxymethyl cellulose (Sigma-Aldrich, CAS: 9004-32-4). After complete dissolution, the medium was sterilized by autoclaving at 121 °C for 15 min. The spore suspension was prepared by filtering the CMC culture medium through a 100 µm membrane (Sefar Nitrex, nylon filter, 100 µm, Dutscher, Issy-les-Moulineaux, France), followed by centrifugation to remove the supernatant. Spores were then resuspended in water. Spore density was determined with a Neubauer counting chamber.

#### 5.2.2. *Aspergillus flavus*

The *A. flavus* E73/NRRL62477 strain, from UMR Toxalim (Toulouse, France), and initially isolated from spices was selected for its strong ability to produce aflatoxins (primarily B1) [41]. The strain was stored on PDA at 4 °C and cultured on the same medium at 25 °C for 10 days until sporulation. Spores were harvested by adding 1 mL of sterile water and scraping the surface of the culture to free the spores. The spore suspension density was further adjusted with a Neubauer counting chamber.

#### 5.2.3. *Penicillium expansum*

The *P. expansum* NRRL 35695 strain (Northern Regional Research Laboratory, Peoria, IL, USA), from UMR Toxalim (Toulouse, Franc) primarily isolated from grape, was selected for its high patulin production [82,83]. The strain was stored on PDA at 4 °C and cultured on the media at 25 °C for 10 days until sporulation. Spores were harvested by adding 1 mL of sterile water and scraping the surface of the culture to release the spores. The spore suspension density was further adjusted with a Neubauer counting chamber.

### 5.3. Preparation of Culture Medium

PDA was prepared at a concentration of 39 g/L. To prepare the Czapek medium (CYA), 1 L of demineralized water is combined with 30 g sucrose (Sigma-Aldrich, for microbiology, ACS reagent, >99.0%, CAS: 57-50-1), 15 g agar (Sigma-Aldrich, Millipore, Saint-Quentin-Fallavier, France CAS: 9002-18-0), 5 g yeast extract (BioKar diagnostics, reference A1202 HA, 500 g), 1 g dipotassium hydrogen phosphate (Fluka, honywell, CAS: 7758-11-4), 0.3 g sodium nitrate (Acros organique, ThermoScientific, CAS: 7631-99-4), 0.05 g potassium chloride (Dominique Dutcher, Cat n°: P2035-500GR), and 0.05 g magnesium sulfate (Honeywell Fluka, CAS: 10034-99-8). Trace elements were prepared by dissolving 0.1 g of iron sulfate (Sigma Life science, Saint-Quentin-Fallavier, France, CAS: 7782-63-0), 0.1 g of zinc sulfate (Fluka chemika, CAS: 7446-20-0), and 0.05 g of copper sulfate (Sigma Life science, CAS: 7758-99-8) in 100 mL of demineralized water. Both culture mediums were sterilized by autoclaving at 120 °C for 15 min. After sterilization, 1 mL of the trace element solution was added to the CYA medium using a sterile syringe equipped with a 0.22 µm polyethersulfone (PES) filter, and the mixture was stirred to ensure homogeneity. Finally, 20 mL of PDA and CYA media were poured into Petri dishes.

### 5.4. In Vitro Evaluation of Antifungal Potential of GCE

#### 5.4.1. GCE Incorporation to Culture Medium

Once the culture medium cooled down to 60 °C, it was mixed with the appropriate weighted quantities of GCE powder in falcon tubes. The mixture was then vortexed for 10 sec to ensure homogeneity. The culture medium/GCE mixture was poured into sterile Petri dishes for solidification at room temperature in sterile condition. The prepared Petri dishes were directly inoculated with the fungal strains. Inoculated plates were placed in an incubator set to 25 °C in the dark.

#### 5.4.2. General Evaluation of Antifungal Activity

In Petri dishes, 10 µL of a spore suspension prepared at 500,000 spores/mL were inoculated. Culture media (PDA or CYA) were supplemented with the extract at 0.25–0.5–1.0–2.5–5.0–10.0–15.0 g/L (see the section on GCE incorporation to culture medium). Control conditions consisted of non-treated culture media and a chemical fungicide (Imazalil^®^, Janssen, France) at 1 g/L mixed with culture media. As described in previous work, fungal growth was monitored by measuring the colony surface from pictures, using ImageJ (1.54p, National Institutes of Health, Bethesda, MD, USA) through 6 to 10 days [41,42,43,44,45,46,47,48,49,50,51,52,53,54,55,56,57,58,59,60,61,62,63,64,65,66,67,68,69,70,71,72,73,74,75,76,77,78,79,80,81,82,83,84].

#### 5.4.3. Sensitivity of the Fungal Targets at Various Physiological Stages

For these experiments, fungi were cultured on cellophane membranes (Le joint Français, Hutchinson) of 1.5 cm or 8 cm, sterilized under UV light for 30 min. Spore suspension was prepared at 500,000 spores/mL. Then, 10 µL of the spore solution was inoculated at the centre of each culture membrane. The Petri dishes were incubated at 25 °C in the dark.

##### Germination Inhibition

Membranes were placed on culture medium, either supplemented with the extract (0.5–2.5–5–10–15–20 g/L for *F. graminearum* and 0.5–5–10–15 g/L for *P. expansum* and *A. flavus*), a commercial fungicide at 1 g/L (positive control) or not supplemented (negative control). To monitor germination, three membranes were observed immediately after inoculation under microscope (Axiolab 5, Zeiss, reference 430037-9011-000) combined with a camera (Toupcam, E3ISPM05000KPA, 60N-C, 2/3”, 0.63×, 426113, Touptek photonics) at various magnifications (10×, 20×, 40×) in bright-field mode. This procedure was repeated every 6 h to monitor spore germination over time. The images were subsequently analyzed using ImageJ software to count the spores and calculate the ratio between the total number of spores and the number of germinated spores at different observation times under the various tested conditions. This ratio enabled the determination of the germination rate.

##### Remanence of Antifungal and Antimycotoxin Activity

Membranes were placed on culture medium, either supplemented with plant extract (0.5–2.5–5–10–15 g/L for *F. graminearum* and 0.5–5–10–15 g/L for *P. expansum* and *A. flavus*), with Imazalil, a commercial fungicide at 1 g/L (positive control) or without antifungal substances (negative control). Three days post-inoculation, six membranes per condition among nine, were transferred to non-treated culture media. The remaining nine membranes were either kept on their initial culture media (6 membranes) or used for mycotoxin analysis (3 membranes) (see section: In vitro evaluation of antimycotoxic potential of GCE). From the time of transfer until the end of the experiment, colony development was monitored for 10 days, with daily macroscopic observation and surface measurement using ImageJ software.

##### Mycelial Stage Inhibition

Membranes were placed on non-treated culture medium and incubated at 25 °C until macroscopic colonies of approximately 1 cm in diameter were visible. Then, membranes were transferred to culture media supplemented with GCE (5.0–10.0–15.0 g/L)*,* a commercial fungicide (Imazalil) at 1 g/L (positive control) or non-treated (negative control). Colony development was monitored for 10 days, with daily photographs and surface measurement using ImageJ software.

### 5.5. In Vitro Evaluation of Antimycotoxigenic Potential of GCE

For each culture condition, mycotoxins extraction was performed at three different time points, typically on days 3, 6, and 9, or as indicated in the Section 2. One half of the agar from the Petri dish, along with the fungal colonies, were transferred into polypropylene (PP) containers (sampling polypropylene container, reference: 35317405, Cloup), and cut into small pieces before extraction with the appropriate solvent. The mass of agar was calculated by subtracting the container mass before and after sampling to adjust the volume of extraction solvent. All solvent volumes mentioned in this section correspond to a 10 g mass of sampled agar along with fungal colony. The containers were then placed on a shaking platform at 250 RPM for 20 min to facilitate the extraction process.

#### 5.5.1. Trichothecenes (TCTBs) Extraction and LC-MS/MS Analysis

The TCTB extraction procedure and LC-MS/MS analysis were previously published and detailed by Pellan et al. 2020 and Dieye et al. 2024 [80,81,82,83,84]. A 30 mL solution of acetonitrile/water/acetic acid (79/20/1, *v*/*v*/*v*) was mixed with half of *F. graminearum* colonies and culture medium in PP containers before homogenization on the shaking platform. The resulting solution containing the extraction solvent and toxins was then diluted at a ratio of 1:50 with ultrapure water/acetic acid (99.5/0.5, *v*/*v*) and filtered through a 0.45 μm cellulose acetate filter into 2 mL amber vials for further LC-MS/MS analysis (Figure S4). TCTB detection and quantification were performed using an Ultra High-Performance Liquid Chromatography (UHPLC, Shimadzu, Tokyo, Japan) coupled with a mass spectrometer (8040, Shimadzu, Tokyo, Japan). LC separation was carried out using a Phenomenex Kinetex XB Column C18 (50 mm × 2 mm; 2.6 μm particles) at 50 °C, with an injection volume of 50 μL. The mobile phase consisted of 0.5% acetic acid in ultra-pure water (solvent A) and 0.5% acetic acid in isopropanol (HPLC MS grade, Sigma, St Louis, MO, USA), (solvent B) at a flow rate of 0.4 mL/min (Table S11). The mass spectrometer was operated in electrospray positive (ESI+) and negative (ESI) ionization mode, and two multiple reaction monitoring (MRM) transitions for each analyte were monitored for quantification (Q) and qualification (q). All data were analyzed using LabSolutions Software (v5.91/2017, Shimadzu, Tokyo, Japan, 2017).

#### 5.5.2. Aflatoxins (AFLAs) Extraction and HPLC Analysis

AFLAs extraction and the HPLC analysis procedure were previously published and detailed by Campos-Avelar et al. (2021) [80]. Half of each Petri dish containing *A. flavus* colonies and agar medium was sampled and mixed with a 33 mL of methanol/formic acid (96/15, *v*/*v*) solution in PP containers. After homogenization, 2 mL of the mixture (solvent, agar, and fungi) was sampled and evaporated for 1 h at 60 °C using an evaporator concentrator (Concentrator plus/Vacufuge^®^plus, Eppendorf). Once evaporation was complete, the extracts were resolubilized in 2 mL of methanol/water (55/45, *v*/*v*), sonicated for 20 min, homogenized, and 1 mL was filtered through a 0.45 µm polytetrafluoroethylene (PTFE) filter into 2 mL amber vials for further HPLC analysis (Figure S5). AFLA detection and quantification were performed using HPLC coupled with a fluorescence detector (Shimadzu RF 20A, Japan) and post-column electrochemical derivatization (Kobra Cell™ R. Biopharm Rône Ltd., Glasgow, UK). *A. flavus* predominantly synthesizes AFB_1_, while other aflatoxins (AFB_2_, AFG_1_, AFG_2_) may be detected at substantially lower concentrations. When present, they exhibit a similar inhibitory response to that observed for AFB1 (Table S2). The operating conditions were as follows: injection volume of 100 µL; C18 reverse-phase HPLC column, Uptisphere type 5 ODB, ODS, 5 µm particle size, 5 ODB, 250 × 4.6 mm, with identical pre-column, thermostatically controlled at 40 °C; isocratic flow rate of 0.8 mL/min (water/methanol (55/45, *v*/*v*) with 119 mg KBr and 350 µL of 4 M nitric acid). Excitation wavelength was 362 nm and emission wavelength was 425 nm. Concentrations were calculated from an AFB_1_ calibration curve established using a standard (25 µg/mL; Biopharm Rhône Ltd., Glasgow, UK). Detection and quantification limits were established at 0.05 and 0.2 ng/mL, respectively.

#### 5.5.3. Patulin (PAT) Extraction and HPLC Analysis

PAT extraction and the HPLC analysis procedure were detailed by Al Riachy [84]. A 25 mL solution of water/acetic acid (99.5/0.5, *v*/*v*) was mixed along with half of *P. expansum* colonies and culture media in PP containers before homogenization on a shaking platform. Then, 2 mL of the solution was sampled, homogenized, and filtered through a 0.45 μm cellulose acetate filter into 2 mL amber vials for HPLC analysis (Figure S6). PAT detection and quantification were performed using HPLC coupled with a UV-vis detector (Shimadzu RF 20A, Japan). Separation was achieved using a LiChrospher C18 column (250 × 4.6 mm, 5 µm) at a flow rate of 1 mL/min, at 35 °C with an injection volume of 100 µL. The mobile phase consisted of ultra-pure water (solvent A) and acetonitrile (solvent B). PAT was detected at 277 nm.

#### 5.5.4. Quantification of Mycotoxins: Data Analysis

Raw data consisted of mycotoxin concentration values expressed in ng/mL (TCTB and AFLA) or µg/mL (PAT). These concentrations were used to calculate the total amount of mycotoxins produced and accumulated in each Petri dish (in ng) and the specific production (in ng/cm^2^), as detailed in Figure S4: TCTB concentration and specific production by *F. graminearum*, Table S10: Multiple reaction monitoring (MRM) parameters for the quantification of DON, 3-ADON, 15-ADON by LC-MS/MS, Table S11: Mobile phase gradient. Solvent (A): water + 0.5% acetic acid (*v*/*v*)/solvent (B): isopropanol + 0.5% formic acid (*v*/*v*) for TCTB analysis, Figure S5: AFLA concentration and specific production by *A. flavus*, and Figure S6: PAT concentration and specific production by *P. expansum*, as well as Table S12: HPLC mobile phase gradient. Water (solvent A)/acetonitrile (solvent B) for patulin analysis.

## Figures and Tables

**Table 1:** Summary of the activity of GCE against *F. graminearum*, *A. flavus* and *P. expansum*. IC_50_ and IC_100_ values (g/L) were reported for radial growth and toxin production; italic ranges indicate the corresponding inhibition level relative to the untreated control (%). For spores, we reported the minimal dose at which no germination was observed under the tested conditions. “Mycelium/colony” indicates the physiological stage at which the fungus was treated, and the related data shown are the maximal growth inhibition measured after transfer to fresh medium; “Remanence” graded the growth and toxin recovery five days after transfer to fresh untreated media (low < 40%, moderate 40–70%, strong > 70%). “Pro-patulin” denotes a stimulatory effect on patulin production; Mycotoxins LOQ are DON = 0.9 µg/cm^2^, AFB1 = 0.001 µg/cm^2^, PAT = 0.08 × 10^−3^ µg/cm^2^, below the analytical limit of quantification. All values were averaged from three independent assays.

Fungi	IC_50_ (g/L)		IC_100_ (g/L)		Spores/Germination		Mycelium/Colony		Remanence	
	Growth	Toxins	Growth	Toxins	Inhibition	Sporicidal	Fungicide	Toxins	Growth	Toxins
*F. graminearum*	2.5 ≈55%	<0.5 ≈88%	2.5–5.0 ≈55–100%	1.0–2.5 ≈94–87%	5.0 and <15.0 ≈72–100%	15.0	not observed 15.0 g/L ≈ 22%	<5.0 <LOQ	moderate	strong
*A. flavus*	2.5–5.0 ≈48–77%	<0.5 ≈98%	>30.0 15.0 g/L ≈ 78%	2.5–5.0 ≈94–100%	>15.0 ≈68%	not observed 15.0 g/L ≈ 68%	not observed 15.0 g/L ≈ 62%	<5.0 ≈99%	moderate	strong
*P. expansum*	1.0–2.5 ≈40–69%	pro-patulin	>30.0 15.0 g/L ≈ 92%	pro-patulin	10.0 ≈21%	not observed 15.0 g/L ≈ 33%	not observed 15.0 g/L ≈ 66%	pro-patulin	low	pro-patulin

**Table 2:** Grapevine cane extract content of the main biochemical families. Results are expressed as % *w*/*w* (weight/weight), indicating that 1% of a given compound corresponds to 1 g of that compound per 100 g of powder cane extract. This unit represents the relative mass fraction of each family of compound within the total extract. * Indicates analytes measured with a method of limited specificity; interfering compounds may affect these values, so quantification is approximate.

Analyte	Content [% *w*/*w*]	SD [% *w*/*w*]	Detection Mode
Water	4.59	1.40	Kar-Fisher-titration method
monosaccharides	1.24	0.81	HPLC-RID
disaccharides	0.70	0.68	HPLC-RID
polysaccharides	14.56	0.38	HPLC-RID
lignin *	70.91	NA	HPLC-UV-vis
polyphenols *	36.90	1.37	folin-Ciocalteu
hydrolysable tannins *	3.98	0.19	absorbance
condensed tannins *	0.41	0.07	absorbance
flavonoids	6.34	NA	ULPC-MS, spectrometry
stilbenoids	21.20	2.56	UPLC-DAD

**Table 3:** Growth- and toxin-inhibition IC_50_ values (µM) of the present GCE and of selected grapevine- and plant-derived stilbenoids in the literature. IC_50_ values are expressed in micromolar (µM) and refer to the concentration that reduced radial growth (“growth” column) or mycotoxin production (“toxin” column) by 50% for *F. graminearum*, *A. flavus*, and *P. expansum*. Stilbenes concentrations from GCE were obtained by HPLC dosage, while bibliographic values were re-calculated from the original data when necessary. <LOQ indicates below the analytical limit of quantification; “-“ not determined. All reported IC_50_ values from the bibliography have a corresponding percentage of inhibition and are reported in parentheses. The reference number in the right column matches the bibliography.

Extracts	Compounds	*F. graminearum* IC_50_ (µM)		Extracts	Compounds	*A. flavus* IC_50_ (µM)		Extracts	Compounds	*P. expansum* IC_50_ (µM)		Reference
		Growth	Toxins			Growth	Toxins			Growth	Toxins	
Grapevine cane extract (GCE) IC_50_ growth: 2.5 g/L IC_50_ toxine: <0.5 g/L	piceid	25.5	1.3	Grapevine cane extract (GCE) IC_50_ growth: 2.5–5.0 g/L IC_50_ toxine: <0.5 g/L	piceid	25.5–61.6	1.3	Grapevine cane extract (GCE) IC_50_ growth: 1.0–2.5 g/L IC_50_ toxine: <30.0 g/L	piceid	5.3–25.5	–	
	piceatanol	13.5	<LOQ		piceatanol	13.5–39.7	<LOQ		piceatanol	6.4–13.5	–	
	trans-resveratrol	138.9	16.4		trans-resveratrol	138.9–233.4	16.4		trans-resveratrol	72.4–138.9	–	
	trans-ε-viniferin	53.2	10.8		trans-ε-viniferin	53.3–94.3	10.8		trans-ε-viniferin	23.9–53.3	–	
	vitisin B	<LOQ	<LOQ		vitisin B	<LOQ–3.8	<LOQ		vitisin B	<LOQ	–	
Pure compound in water: 0.1 g/L	piceatanol	>410.0 (≈no inhibition)	–	Pure compound in water: 0.1 g/L	piceatanol	>410.0 (≈23% inhibition)	–	Pure compound in water: 0.1 g/L	piceatanol	>410.0 (≈no inhibition)	–	[28]
Sauvignon blanc canes in H_2_O/EtOH, 95.5/0.5, *v*/*v* IC50: >0.1 g/L	piceatanol	>8.1	8.1	Pure compound in water: 0.003 g/L	trans-resveratrol	13.2 (≈no inhibition)	13.2 (≈47% inhibition)	Pure compound in phosphate buffer/NaoH, 9:1, *v*/*v*): 5.0 g/L	trans-resveratrol	21,900 (≈7.2% inhibition)	21,900 (+40%)	[50,51,52]
	trans-resveratrol	>43.2	43.2									
	trans-ε-viniferin	>110.9 (<50% inhibition)	110.9 (<36% inhibition)									
Tannat canes in H_2_O/EtOH, 95.5:0.5, *v*/*v* IC50: >0.09 g/L	piceatanol	4.9	6.9									[50]
	trans-resveratrol	31.0	43.6									
	trans-ε-viniferin	61.2 (50% inhibition)	86.0 (69% inhibition)									
Merlot woods in H_2_O/EtOH, 95.5/0.5, *v*/*v* IC50: 0.07 g/L	trans-resveratrol	73.7	139.5									[50]
	trans-ε-viniferin	49.8 (50% inhibition)	94.4 (81% inhibition)									
Tannat wood in H_2_O/EtOH, 95.5/0.5, *v*/*v* IC50: 0.07 g/L	trans-resveratrol	16.8	28.8									[50]
	trans-ε-viniferin	44.7 (50% inhibition)	76.6 (80% inhibition)									

**Table 4:** HPLC mobile phase gradient. Solvent (A): water + 0.1% formic acid (*v*/*v*)/solvent (B): acetonitrile + 0.1% formic acid (*v*/*v*) for GCE analysis.

Time (min)	Solvent B (%)
0.0	5
3.0	30
9.2	40
9.4	100
10.5	100
10.7	5
11.3	5

## Data Availability

The original contributions presented in this study are included in the article and Supplementary Materials. Further inquiries can be directed to the corresponding authors.

## Supplementary Materials

The following supporting information can be downloaded at: https://www.mdpi.com/article/10.3390/toxins17090454/s1. Figure S1: *F. graminearum* kinetic growth on CYA medium supplemented with grapevine cane extract directly incorporated into the agar; Figure S2: *A. flavus* kinetic growth on PDA medium supplemented with grapevine cane extract directly incorporated into the agar; Figure S3: *Penicillium expansum* kinetic growth on PDA medium supplemented with grapevine cane extract directly incorporated into the agar; Table S1: Quantification of mycotoxins produced by *F. graminearum*, *A. flavus*, and *P. expansum* on culture media supplemented with grapevine cane extract; Table S2: Quantification of aflatoxins produced by *Aspergillus flavus* on culture media supplemented with grapevine cane extract; Table S3: Quantification of trichothecenes (DON, 15-ADON, and 3-ADON) produced by *F. graminearum* on CYA supplemented with cane extract; Table S4: Quantification of trichothecenes (DON, 15-ADON, and 3-ADON) produced by *F. graminearum* at a mycelial stage on CYA supplemented with cane extract; Table S5: Quantification of aflatoxin B1 (AFB1) produced by *A. flavus* on PDA supplemented with cane extract; Table S6: Quantification of aflatoxin B1 (AFB1) produced by *A. flavus* at a mycelial stage on PDA supplemented with cane extract; Table S7: Quantification of patulin (PAT) produced by *P. expansum* on PDA supplemented with cane extract; Table S8: Quantification of patulin (PAT) produced by *P. expansum* at a mycelial stage on PDA supplemented with cane extract; Table S9: Main stilbenoid compounds identified in the grapevine cane extract; Figure S4: Quantification of TCTB concentration and specific production by *F. graminearum*; Table S10: Multiple reaction monitoring (MRM) parameters for the quantification of DON, 3-ADON, 15-ADON by LC-MS/MS; Table S11: Mobile phase gradient. solvent (A): water + 0.5% acetic acid (*v*/*v*)/solvent (B): isopropanol + 0.5% formic acid (*v*/*v*) for TCTB analysis; Figure S5: Quantification of AFLA concentration and specific production by *A. flavus*; Figure S6: Quantification of PAT concentration and specific production by *P. expansum*; Table S12: Mobile phase gradient. Water (solvent A)/acetonitrile (solvent B) for patulin analysis.
