# Supplementary material for: In Vitro Screening of the Antifungal and Antimycotoxin Effects of a Stilbenoids-Riche Grapevine Cane Extract on Fusarium graminearum, Aspergillus flavus and Penicillium expansum
Source: Toxins (Basel). 2025 Sep 9;17(9):454. doi: 10.3390/toxins17090454 (PMC12474358; doi:10.3390/toxins17090454)
Supplement: Supplementary file 1 [file toxins-17-00454-s001.zip › toxins-3806215-supplementary.pdf]

Supplementary Materials:

1. **Growth and mycotoxin inhibition by GCE on *F. graminearum*, *A. flavus*, and *P. expansum***

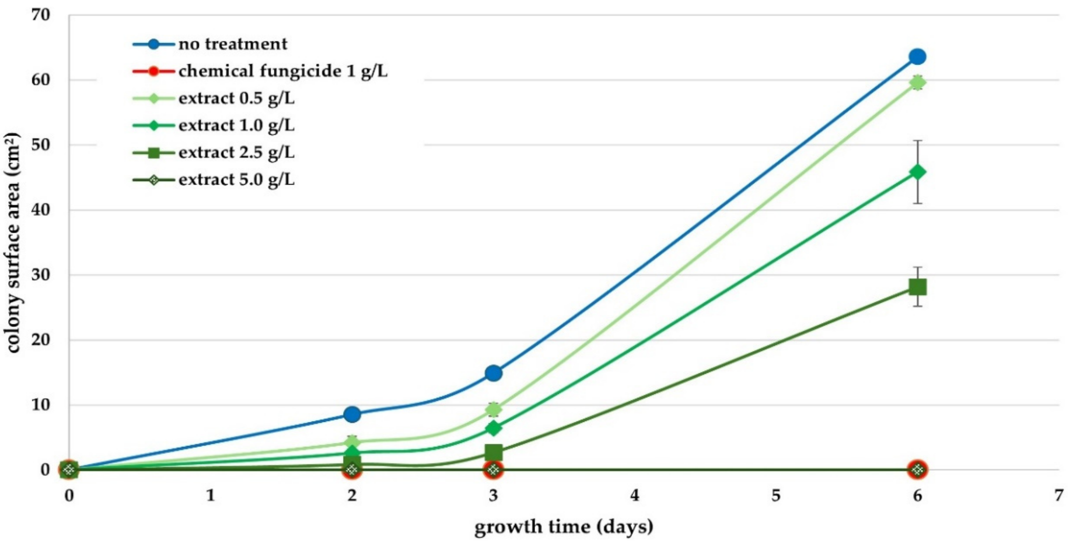

Figure S1: *F. graminearum* kinetic growth on CYA medium supplemented with grapevine cane extract directly incorporated into the agar. Surface areas occupied by *F. graminearum* in the presence of the extract at four concentrations (0.5 g/L. 1.0 g/L. 2.5 g/L. and 5.0 g/L. shown in varying shades of green) were compared to non-treated media (optimal growth conditions, in blue) and media supplemented with a chemical fungicide at 1 g/L (commercial fungicide, in red). Surface areas were measured daily over 6 days post-inoculation. ImageJ software was used to evaluate the fungal surface area. Each point represented the average of three replicates.

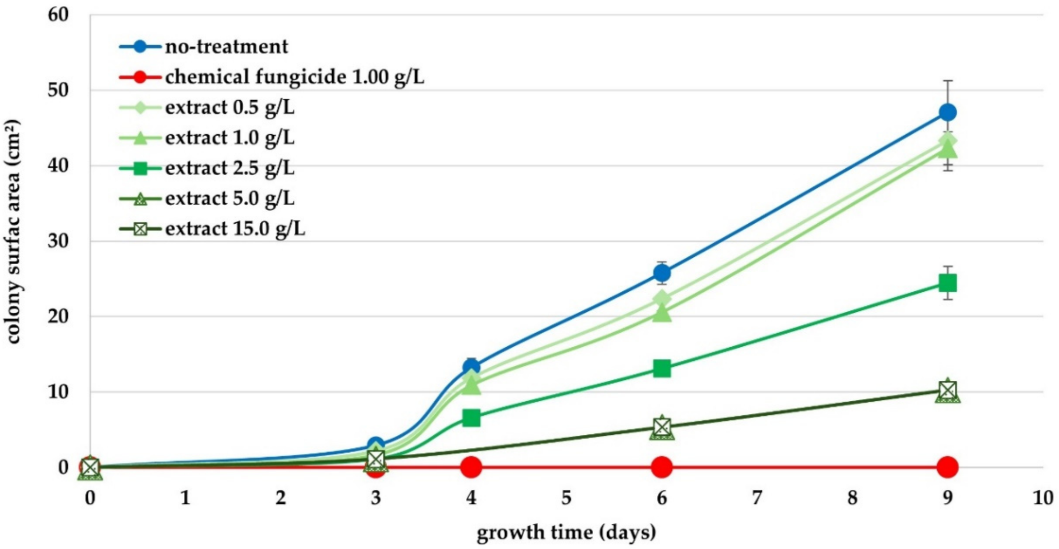

Figure S2: *A. flavus* kinetic growth on PDA medium supplemented with grapevine cane extract directly incorporated into the agar. Surface areas occupied by *A. flavus* in the presence of the extract at five concentrations (0.5 g/L. 1.0 g/L. 2.5 g/L. 5.0 g/L. and 15.0 g/L. shown in varying shades of green) were compared to non-treated media (optimal growth conditions. in blue) and media supplemented with a chemical fungicide at 1 g/L (commercial fungicide. in red). Surface areas were measured daily for nine days post-inoculation. ImageJ software was used to evaluate the fungal surface area. Each point represented the average of three replicates.

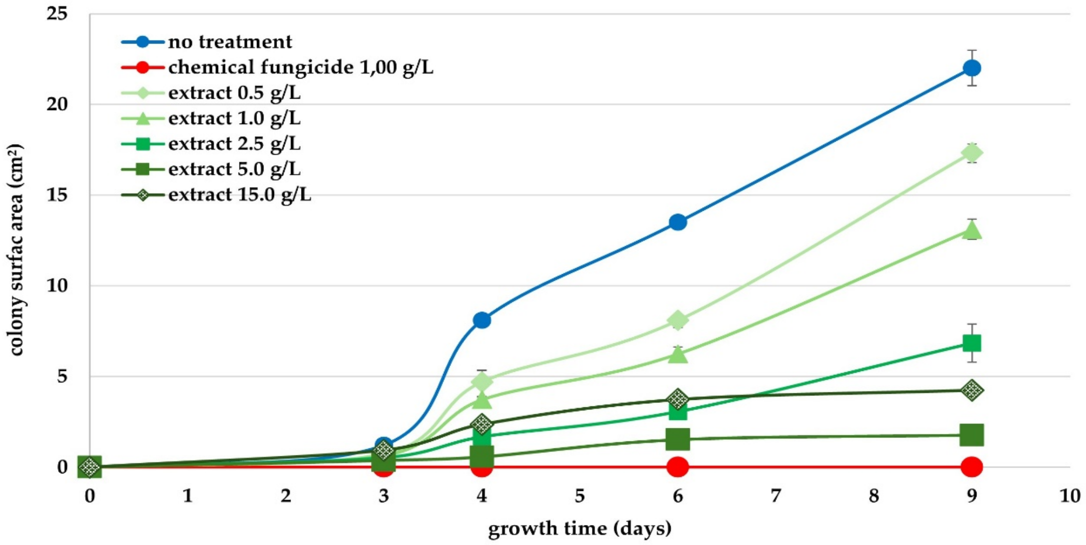

Figure S3: *Penicillium expansum* kinetic growth on PDA medium supplemented with grapevine cane extract directly incorporated into the agar. Surface areas occupied by *P. expansum* in the presence of the extract at five concentrations (0.5 g/L. 1.0 g/L. 2.5 g/L. 5.0 g/L. and 15.0 g/L. shown in varying shades of green) were compared to non-treated media (optimal growth conditions. in blue) and media supplemented with a chemical fungicide at 1 g/L (commercial fungicide. in red). Surface areas were measured daily for nine days post-inoculation. ImageJ software was used to evaluate the fungal surface area. Each point represented the average of three replicates.

Table S1. Quantification of mycotoxins produced by *F. graminearum*, *A. flavus*, and *P. expansum* on culture media supplemented with grapevine cane extract. Absolute toxin levels (µg) were measured in the culture media after 6 or 9 days of incubation at 25 °C. Specific production (µg/cm²) was calculated as the ratio between the toxin quantity (µg) and the corresponding fungal colony surface area (cm²). All values are representative of three independent biological replicates followed by the standard deviation Trichothecenes (DON, 15-ADON, and 3-ADON) were produced by *F. graminearum*, aflatoxin B1 (AFB1) by *A. flavus*, and patulin (PAT) by *P. expansum*. The impact of grapevine cane extract was evaluated at different concentrations (0.25, 0.5, 1.0, 2.5, 5.0, and 15.0 g/L) and compared to non-treated control media (optimal growth condition). Toxin quantification was performed using HPLC-MS for trichothecenes, HPLC with KOBRA-cell detection for AFB1, and HPLC for PAT.

Mycotoxin (coumpound name)		plant extract concentration (g/L)	quantity (µg)		Specific production (µg/cm²)	
			day 6	day 9	day 6	day 9
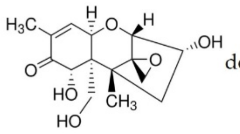	deoxynivalenol (DON)	no-treatment	237.60 ± 35.91		3.74 ± 0.56	
		Extract 0.25 g/L	155.59 ± 16.26		2.55 ± 0.27	
		Extract 0.5 g/L	26.64 ± 5.37		0.45 ± 0.09	
		Extract 1.0 g/L	9.19 ± 2.70		0.20 ± 0.06	
		Extract 2.5 g/L	10.27 ± 5.86		0.50 ± 0.11	
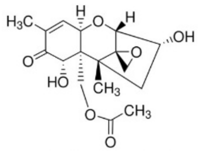	15 acetyl-deoxynivalenol (15-ADON)	no-treatment	356.27 ± 29.13		5.60 ± 0.46	
		Extract 0.25 g/L	228.08 ± 29.52		3.74 ± 0.48	
		Extract 0.5 g/L	45.96 ± 10.56		0.77 ± 0.18	
		Extract 1.0 g/L	0.00 ± 0.00		0.00 ± 0.00	
		Extract 2.5 g/L	0.00 ± 0.00		0.00 ± 0.00	

Mycotoxin (coumpound name)		plant extract concentration (g/L)	quantity (μg)	Specific production (μg/cm²)
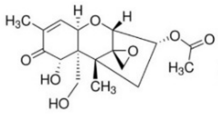	3 acetyl- leoxynivalenol (3-ADON)	no-treatment	72.51 ± 5.90	1.14 ± 0.09
		Extract 0.25 g/L	54.31 ± 2.14	0.89 ± 0.04
		Extract 0.5 g/L	2.60 ± 1.06	0.04 ± 0.02
		Extract 1.0 g/L	0.00 ± 0.00	0.00 ± 0.00
		Extract 2.5 g/L	0.44 ± 0.38	0.02 ± 0.01
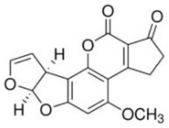	Aflatoxine B1 (AFB1)	no-treatment	119.16 ± 26.08	2.53 ± 0.55
		Extract 0.25 g/L	6.27 ± 1.40	0.14 ± 0.03
		Extract 0.5 g/L	3.73 ± 1.54	0.07 ± 0.01
		Extract 1.0 g/L	2.06 ± 0.36	0.05 ± 0.01
		Extract 2.5 g/L	3.73 ± 1.54	0.15 ± 0.06
		Extract 5.0 g/L	0.00 ± 0.00	0.00 ± 0.00
		Extract 15.0 g/L	0.00 ± 0.00	0.00 ± 0.00
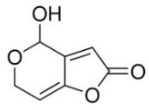	Patulin (PAT)	no-treatment	30.14 ± 5.71	1.37 ± 0.26
		Extract 0.25 g/L	21.88 ± 0.44	1.01 ± 0.02
		Extract 0.5 g/L	23.52 ± 5.01	1.36 ± 0.29
		Extract 1.0 g/L	21.36 ± 4.66	1.63 ± 0.35
		Extract 2.5 g/L	22.15 ± 5.66	3.24 ± 0.83
		Extract 15.0 g/L	20.32 ± 0.40	4.75 ± 0.09
		Extract 5.0 g/L	19.41 ± 0.68	8.05 ± 0.28

2. ***A. flavus*** **E73/NRRL62477 strain is an AFB1-dominant producer**

Table S2. Quantification of aflatoxins produced by *Aspergillus flavus* on culture media supplemented with grapevine cane extract. Absolute toxin levels (ng/mL) in vials from culture media after 6 or 9 days of incubation at 25 °C. Four aflatoxins were measured: aflatoxin B1 (AFB1), aflatoxin B2 (AFB2), aflatoxin G1 (AFG1), aflatoxin G2 (AFG2). The impact of grapevine cane extract was evaluated at different concentrations (1.0, 5.0, and 15.0 g/L) and compared to non-treated control media (optimal growth condition). Aflatoxin quantification was performed using HPLC with KOBRA-cell. Values are representative of six independent biological replicates.

Mycotoxin (coumpound name)		plant extract concentration (ng/L)	concentration (ng/mL)	
			day 6	day 9
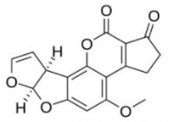	Aflatoxin B1 (AFB1)	no-treatment	287.11 ± 54.22	682.63 ± 99.46
		Extract 1.0 g/L	3.81 ± 0.67	10.93 ± 1.58
		Extract 5.0 g/L	< LOQ	1.72 ± 0.00
		Extract 15.0 g/L	< LOQ	< LOQ
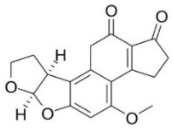	Aflatoxin B2 (AFB2)	no-treatment	3.84 ± 0.11	9.18 ± 1.18
		Extract 1.0 g/L	0.07 ± 0.13	0.25 ± 1.58
		Extract 5.0 g/L	< LOQ	0.07 ± 0.12
		Extract 15.0 g/L	0.23 ± 0.00	0.07 ± 0.12
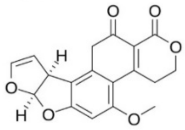	Aflatoxin G1 (AFG1)	no-treatment	0.18 ± 0.26	0.65 ± 0.06
		Extract 1.0 g/L	< LOQ	< LOQ
		Extract 5.0 g/L	< LOQ	< LOQ
		Extract 15.0 g/L	< LOQ	0.20 ± 0.17
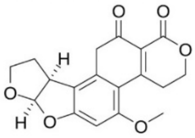	Aflatoxin G2 (AFG2)	no-treatment	3.00 ± 0.16	6.21 ± 1.06
		Extract 1.0 g/L	< LOQ	0.22 ± 0.19
		Extract 5.0 g/L	< LOQ	< LOQ
		Extract 15.0 g/L	< LOQ	< LOQ

A. flavus E73 (NRRL 62477) predominantly produces AFB1, with AFB2, AFG1, and AFG2 either not detected or detected at much lower concentrations as shown in Table S2 and consistent with the bibliography [41–42–43].

3. **Inhibition through fungal life cycle: TCTB production by *F.* graminearum before and after transfer**

3.1. DON and 15-ADON production before dans after transfer from treated to non-treated media

Table S3. Quantification of trichothecenes (DON, 15-ADON, and 3-ADON) produced by *F. graminearum* on CYA supplemented with cane extract. Absolute toxin levels (µg) were measured in the culture media after 5 (transfer day), 8 and 10 days of incubation at 25 °C. Specific production (µg/cm²) was calculated as the ratio between the toxin quantity (µg) and the corresponding fungal colony surface area (cm²). The impact of cane extract was evaluated at different concentrations (0.5, 5.0, 10.0, and 15.0 g/L) and compared to non-treated control media (optimal growth condition). trichothecenes quantification was performed using HPLC-MS. In this experiment no 3-ADON was produced by *F. graminearum*.

mycotoxins	conditions	quantity (µg)			Specific production (µg/cm²)		
		day 5	day 8	day10	day 5	day 8	day10
deoxynivalenol (DON)	no-treatment - NT	145.8 ± 40.3	198.1 ± 35.1	643.2 ± 8.8	2.7 ± 0.7	3.1 ± 0.6	10.2 ± 0.1
	no-treatment - T	N.D.	245.8 ± 47.8	303.5 ± 41.08	N.D.	4.3 ± 0.8	5.2 ± 0.7
	Extract 5.0 g/L - T	N.D.	N.D.	19.2 ± 3.9	N.D.	N.D.	0.4 ± 0.1
	Extract 10.0 g/L - T	N.D.	N.D.	< LOQ	N.D.	N.D.	< LOQ
	Extract 15.0 g/L - T	N.D.	N.D.	N.D.	N.D.	N.D.	N.D.
15 acetyl-deoxynivalenol (15-ADON)	no-treatment - NT	119.5 ± 52.3	182.7 ± 9.6	226.9 ± 47.0	2.2 ± 1.0	2.9 ± 0.2	3.60 ± 0.7
	no-treatment - T	N.D.	249.5 ± 54.3	303.5 ± 54.3	N.D.	3.3 ± 1.0	3.22 ± 0.1
	Extract 5.0 g/L - T	N.D.	N.D.	< LOQ	N.D.	N.D.	< LOQ
	Extract 10.0 g/L - T	N.D.	N.D.	< LOQ	N.D.	N.D.	< LOQ
	Extract 15.0 g/L - T	N.D.	N.D.	N.D.	N.D.	N.D.	N.D.

3.2. DON, 3-ADON, 15-ADON production before dans after transfer from non-treated to treated media

Table S4. Quantification of trichothecenes (DON, 15-ADON, and 3-ADON) produced by *F. graminearum* at a mycelial stage on CYA supplemented with cane extract. Absolute toxin levels (µg) were measured in the culture media after 6 days of incubation at 25°C. Specific production (µg/cm²) was calculated as the ratio between the toxin quantity (µg) and the corresponding fungal colony surface area (cm²). The impact of cane extract was evaluated at either fungistatic or fungicide concentrations (5.0, and 15.0 g/L) and compared to non-treated control (optimal growth condition). trichothecenes quantification was performed using HPLC-MS.

mycotoxins	conditions	quantity (µg)	Specific production (µg/cm²)
deoxynivalenol (DON)	no-treatment - T	134.9 ± 23.5	2.1 ± 0.4
	Extract 5.0 g/L - T	< LOQ	< LOQ
	Extract 15.0 g/L - T	< LOQ	< LOQ
15 acetyl-deoxynivalenol (15-ADON)	no-treatment - T	153.2 ± 25.1	2.4 ± 0.4
	Extract 5.0 g/L - T	< LOQ	< LOQ
	Extract 15.0 g/L - T	< LOQ	< LOQ
3 acetyl-deoxynivalenol (3-ADON)	no-treatment - T	112.2 ± 54.32	1.8 ± 0.9
	Extract 5.0 g/L - T	< LOQ	< LOQ
	Extract 15.0 g/L - T	< LOQ	< LOQ

4. **Inhibition through fungal life cycle: AFB1 production by *A. flavus* before and after transfer**

4.1. AFB1 production before dans after transfer from treated to non-treated media

Table S5. Quantification of aflatoxin B1 (AFB1) produced by *A. flavus* on PDA supplemented with cane extract. Absolute toxin levels (µg) was measured in the culture media after 5 (transfer day), 8 and 11 days of incubation at 25 °C. Specific production (µg/cm²) was calculated as the ratio between the toxin quantity (µg) and the corresponding fungal colony surface area (cm²). The impact of cane extract was evaluated at different concentrations (5.0, 10.0, and 15.0 g/L) and compared to non-treated control media (optimal growth condition). AFB1 quantification was performed using HPLC combined to a Kobra-cell.

mycotoxins	conditions	quantity (µg)			Specific production (µg/cm²)		
		day 5	day 8	day11	day 5	day 8	day11
Aflatoxin B1 (AFB1)	no-treatment - NT	19.7 ± 11.1	32.4 ± 18.8	89.6 ± 20.4	1.4 ± 0.8	0.9 ± 0.5	1.8 ± 0.4
	no-treatment - T	N.D.	60.1 ± 12.8	197.6 ± 34.9	N.D.	2.8 ± 0.8	5.0 ± 0.8
	Extract 5.0 g/L - T	< LOQ	0.3 ± 0.2	17.5 ± 12.7	< LOQ	0.02 ± 0.01	0.3 ± 0.08
	Extract 10.0 g/L - T	< LOQ	0.2 ± 0.04	47.2 ± 14.1	< LOQ	0.01 ± 0.01	1.5 ± 0.2
	Extract 15.0 g/L - T	< LOQ	0.1 ± 0.1	12.0 ± 8.2	< LOQ	0.01 ± 0.01	0.2 ± 0.08

4.2. AFB1 production before dans after transfer from non-treated to treated media

Table S6. Quantification of aflatoxin B1 (AFB1) produced by *A. flavus* at a mycelial stage on PDA supplemented with cane extract. Absolute toxin levels (µg) were measured in the culture media after 8 days of incubation at 25°C. Specific production (µg/cm²) was calculated as the ratio between the toxin quantity (µg) and the corresponding fungal colony surface area (cm²). The impact of cane extract was evaluated at different concentrations (5.0, and 15.0 g/L) and compared to non-treated control (optimal growth condition). AFB1 quantification was performed using HPLC combined to a Kobra-cell.

mycotoxins	conditions	quantity (µg)	Specific production (µg/cm²)
Aflatoxin B1 (AFB1)	no-treatment - T	54.3 ± 4.0	1.4 ± 0.1
	Extract 5.0 g/L - T	0.1 ± 0.06	0.01 ± 0.00
	Extract 15.0 g/L - T	< LOQ	< LOQ

5. **Inhibition through fungal life cycle: PAT production by *P. expansum* before and after transfer**

5.1. PAT production before dans after transfer from treated to non-treated media

Table S7. Quantification of patulin (PAT) produced by *P. expansum* on PDA supplemented with cane extract. Absolute toxin levels (µg) was measured in the culture media after 5 (transfer day), 8 and 11 days of incubation at 25 °C. Specific production (µg/cm²) was calculated as the ratio between the toxin quantity (µg) and the corresponding fungal colony surface area (cm²). The impact of cane extract was evaluated at different concentrations (5.0, 10.0, and 15.0 g/L) and compared to non-treated control media (optimal growth condition). PAT quantification was performed using HPLC.

mycotoxins	conditions	quantity (µg)			Specific production (µg/cm²)		
		day 5	day 8	day11	day 5	day 8	day11
Patulin (PAT)	no-treatment - NT	1416.1 ± 606.3	1684.6 ± 501.3	3119.7 ± 170.1	170.0 ± 72.8	77.4 ± 23.0	132.2 ± 7.2
	no-treatment - T	N.D.	3912.3 ± 812.3	2802.6 ± 722.9	N.D.	277.0 ± 76.4	227.6 ± 74.6
	Extract 5.0 g/L - T	1645.2 ± 77.2	2627.4 ± 454.6	6602.7 ± 660.1	389.9 ± 18.3	435.1 ± 27.6	652.6 ± 39.3
	Extract 10.0 g/L - T	1598.6 ± 195.5	4714.9 ± 1180.0	6836.1 ± 969.2	408.9 ± 50.0	529.3 ± 67.2	657.6 ± 67.3
	Extract 15.0 g/L - T	1618.0 ± 113.0	3210.8 ± 113.7	4948.0 ± 567.9	573.7 ± 40.1	671.4 ± 40.1	807.9 ± 56.0

5.2. PAT production before dans after transfer from non-treated to treated media

Table S8. Quantification of patulin (PAT) produced by *P. expansum* at a mycelial stage on PDA supplemented with cane extract. Absolute toxin levels (µg) were measured in the culture media after 8 days of incubation at 25°C. Specific production (µg/cm²) was calculated as the ratio between the toxin quantity (µg) and the corresponding fungal colony surface area (cm²). The impact of cane extract was evaluated at different concentrations (5.0, and 15.0 g/L) and compared to non-treated control (optimal growth condition). PAT quantification was performed using HPLC.

mycotoxins	conditions	quantity (µg)	Specific production (µg/cm²)
Patulin (PAT)	no-treatment - T	3771.5 ± 709.6	238.7 ± 44.9
	Extract 5.0 g/L - T	1815.0 ± 115.3	313.6 ± 19.9
	Extract 15.0 g/L - T	2512.1 ± 206.6	597.0 ± 49.1

6. Detailed biochemical composition in stilbenoïd from GCE

Table S9: main stilbenoïd compounds identified in the grapevine cane extract. The extract was prepared at 5 g/L in ethanol/water, 50:50, v/v. Compounds were identified by cross comparison between bibliography data and mass spectrometry results. Analysis led to the detection and annoation of 21 stilbenes.

Compound group	Compound annotation	Chemical formula	MW (g/mol)	[M-H]- Precursor Ion	Main MS/MS fragments (m/z)
Stilbene monomer	Resveratrol C-hexoside	C20 H22 O8	390,1308	389,1235	**241**, 269, 251, 163, 199
Stilbene dimer	Oxidized resveratol dimer	C28 H24 O7	472,1513	471,1442	**255**, 121, 187, 349, 211471,1442
Stilbene monomer	trans-ε-piceid	C20 H22 O8	390,1305	389,1232	**227**
Stilbene dimer	Oxidized resveratol dimer	C28 H24 O7	472,1513	471,1444	**121**, 349, 243
Stilbene dimer	Oxidized resveratol dimer	C28 H24 O7	472,1513	471,1442	**121**, 265, 349
Stilbene dimer	Ampelopsin A	C28 H22 O7	470,1357	469,1283	**451,** 423 345, 357 317
Stilbene monomer	trans-piceatannol	C14 H12 O4	244,0729	243,0656	**159,** 201, 243, 175, 173
Stilbene dimer	Oxidized resveratol dimer	C28 H24 O7	472,1515	471,1444	**121**, 255, 349
Stilbene dimer	Ampelopsin F	C28 H22 O6	454,1407	453,1337	**359,** 93, 317, 289, 265
Stilbene dimer	Pallidol	C28 H22 O6	454,1407	453,1334	**265,** 93, 359, 221

Stilbene dimer	Ampelopsine D	C28 H22 O6	454,1407	453,1337	**289,** 359, 343, 291, 453, 317
Stilbene monomer	trans-resveratrol	C14 H12 O3	228,0782	227,071	**143,** 185, 227, 183, 159
Stilbene dimer	cis-δ-viniferine	C28 H22 O6	454,1407	453,1336	**289,** 359, 93, 317, 281, 341
Stilbene dimer	Ampelopsin B	C28 H22 O6	454,1407	453,1336	**359,** 347, 240, 93, 317
Stilbene tetramer	Hopeaphenol	C56 H42 O12	906,2664	905,2592	**359,** 265, 451, 611, 717, 811
Stilbene tetramer	Isohopeaphenol	C56 H42 O12	906,2664	905,2592	**359,** 265, 357, 451, 717, 611
Stilbene tetramer	Ampelopsin H	C56 H42 O12	906,2664	905,2593	**717,** 611, 811, 490
Stilbene dimer	trans-ε-viniferine	C28 H22 O6	454,1405	453,1333	**225,** 347, 369, 93, 253
Stilbene trimer	(E)-miyabenol C	C42H32O9	680,2041	679,1967	NA
Stilbene tetramer	Vitisine A	C56 H42 O12	906,2663	905,2598	**359,** 357, 439, 799, 705, 649, 451, 241
Stilbene tetramer	Vitisine B	C56 H42 O12	906,2664	905,259	**799,** 359, 905, 439, 781

7. Detailed methodology to extract and quantify mycotoxins

7.1. TCTB extraction and quantification from Petri Dish

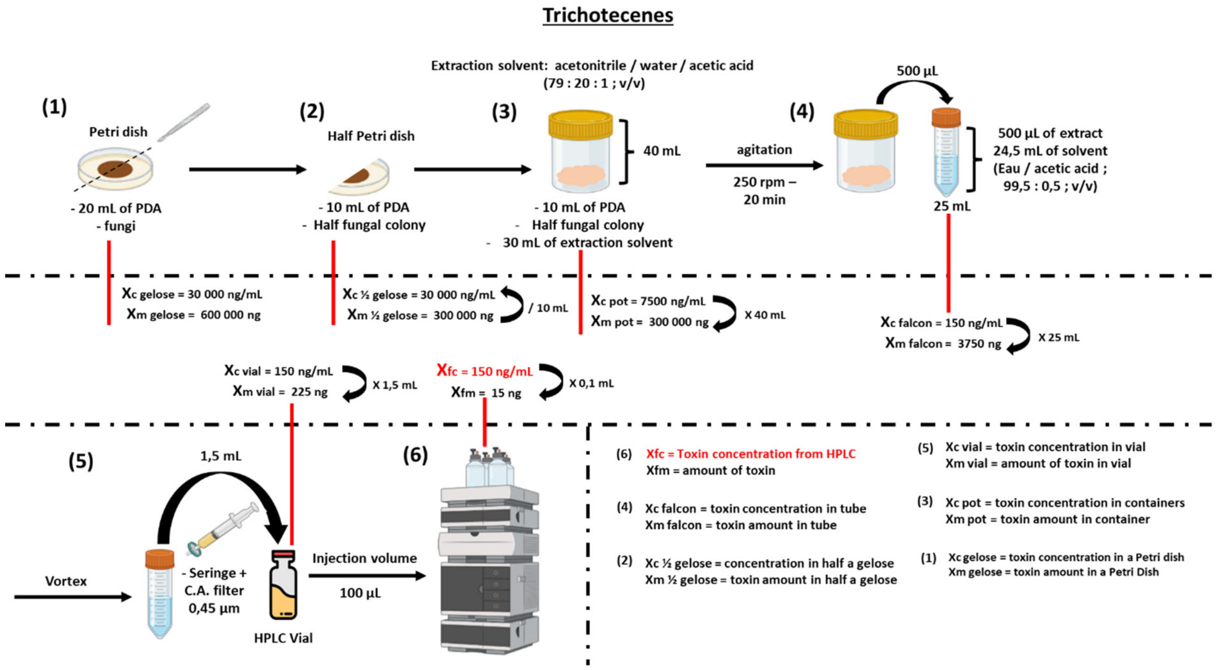

Figure S4: TCTB concentration and specific production by *F. graminearum*. All the steps for TCTB extraction are described: (1) → (2) half a gelose along with the fungal colony is sampled in a PP container, (3) agar medium and fungal colony are mix with the extraction solvent and homogenize, (4) 500 µL are sampled from PP containers and diluted in another solvent (ratio 1:50), (5) 1.5 mL are sampled from the tube into an amber vial though a filter, (6) detection and quantification by LC-MS. Raw data obtained from the LC-MS are TCTB concentrations quantify from the 100 µL previously injected. As an example of raw data, a concentration of 150 ng/mL was written in this scheme (red).

Table S10: Multiple reaction monitoring (MRM) parameters for the quantification of DON, 3-ADON, 15-ADON by LC-MS/MS. The table indicates the polarity mode used, the quantifier (MRM Q) and qualifier (MRM q) ion transitions, along with their corresponding collision energies (CE, in electron volts, eV). These transitions were optimized to ensure accurate detection of each analyte.

Mycotoxins	Polarity	MRM Q	CE MRM Q(eV)	MRM q	CE MRM q (eV)
DON	-	397.2 > 59	25	397.2 > 307.1	16
3ADON	-	397.2 > 59	25	397.2 > 307.1	16
15ADON	+	338.9 > 137.1	-19	338.9 > 297.2	-14

Table S11: mobile phase gradient. solvent (A): water + 0,5 % acetic acid (v/v) / solvent (B): isopropanol + 0,5 % formic acid (v/v) for TCTB analysis.

Time (min)	solvent B (%)
0.01	10
1.50	55
3.50	85
4.00	80
4.01	2
11.00	2

7.2. Aflatoxins extraction and quantification from Petri Dish

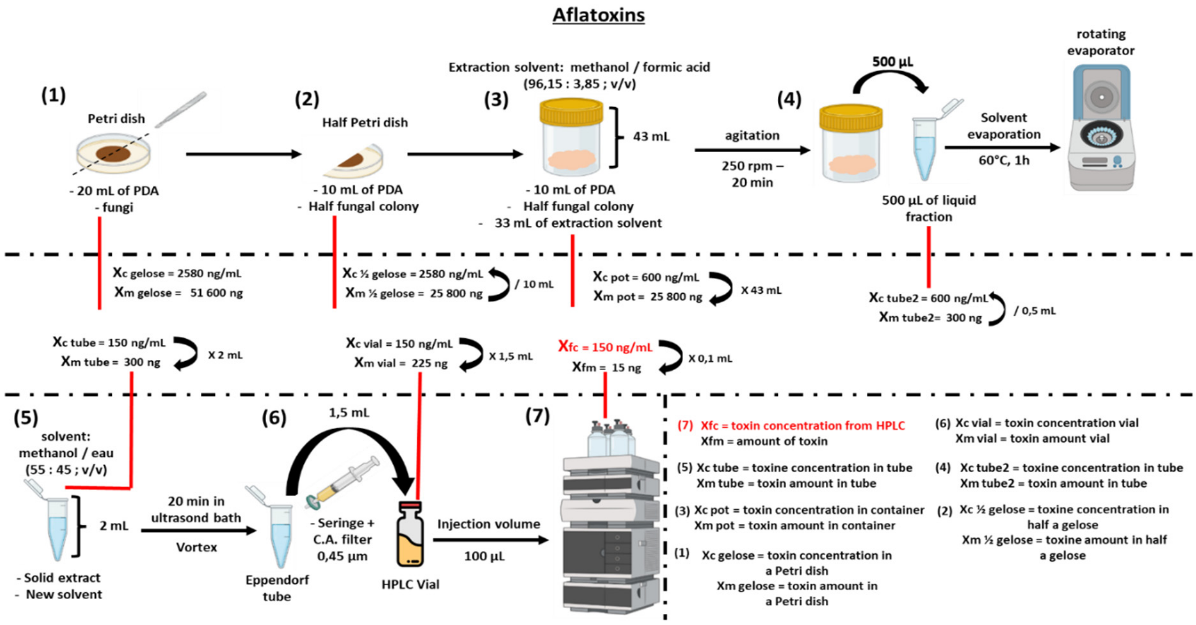

Figure S5: AFLA concentration and specific production by *A. flavus*. All the steps for AFLA extraction are described: (1) → (2) half a gelose along with the fungal colony is sampled in a PP container, (3) agar medium and fungal colony are mix with the extraction solvent and homogenize, (4) 500 µL are sampled from PP containers into a tube and evaporate using a rotating evaporator, (5) sample are resolubilize, sonicate and homogenise, (6) 1.5 mL are sampled from the tube into an amber vial though a filter, (6) detection and quantification by HPLC. Raw data obtained from the HPLC are AFLA concentrations quantify from the 100 µL previously injected. As an example of raw data, a concentration of 150 ng/mL was written in this scheme (red).

7.3. Patulin extraction and quantification from Petri Dish

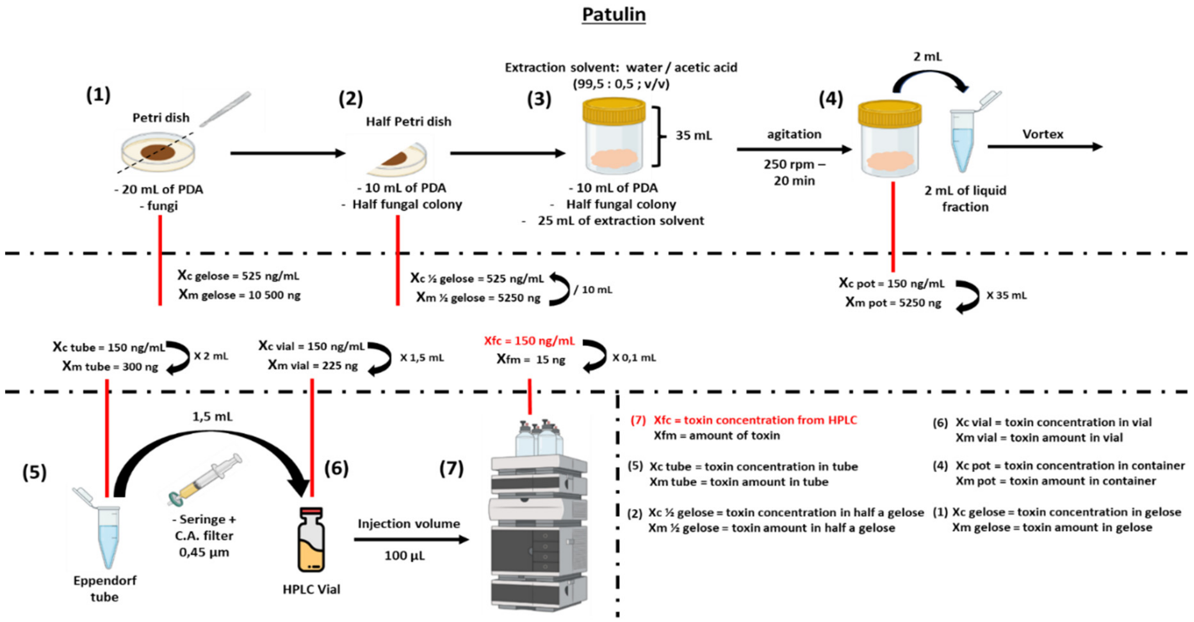

Figure S6: PAT concentration and specific production by *P. expansum*. All the steps for PAT extraction are described: (1) → (2) half a gelose along with the fungal colony is sampled in a PP container, (3) agar medium and fungal colony are mix with the extraction solvent and homogenize, (4) → (5) 2 mL are

sampled from PP containers into a tube, (6) 1.5 mL are sampled from the tube into an amber vial though a filter, (7) detection and quantification by HPLC. Raw data obtained from the HPLC are PAT concentrations quantify from the 100 µL previously injected. As an example of raw data, a concentration of 150 ng/mL was written in this scheme (red).

Table S12: HPLC mobile phase gradient. Water (solvent A) / acetonitrile (solvent B) for patulin analysis

Time (min)	solvent B (%)
0.0	5
16.0	2
20.0	60
26.0	5
